# Participating in a Digital-History Project Mobilizes People for Symbolic Justice and Better Intergroup Relations Today

**DOI:** 10.1177/09567976251331040

**Published:** 2025-04-17

**Authors:** Ruth Ditlmann, Berenike Firestone, Oguzhan Turkoglu

**Affiliations:** 1 Hertie School; 2 WZB Berlin Social Science Center

**Keywords:** past atrocities, mobilization, symbolic justice, intergroup relations, efficacy

## Abstract

Awareness of past atrocities is widely seen as critical for restoring justice and building resilient democracies. Going beyond information provision, an increasing number of memorial sites, museums, and historical archives offer opportunities for public participation. Yet little empirical evidence exists on the impact of participation in the collective remembrance of past atrocities. Two experimental studies, a field-in-the-lab study with 552 university students in Germany and an online randomized control trial with 900 digital workers in Germany, showed that participating in a large-scale, digital-history project about Nazi persecution increased peoples’ collective-action intentions for further commemoration activities and for activities that strengthen intergroup relations today. These effects persisted for 2 weeks. The findings suggest that digital-history projects can motivate collective action that is critical for symbolic justice and positive intergroup relations, thus contributing to well-functioning, pluralistic democracies.

Awareness of past atrocities is widely seen as critical for restoring justice and building resilient democracies (Balcells & Voytas, 2023; Balcells et al., 2022; UNESCO, 2017). For the remembrance of past atrocities to have this desired impact, it cannot merely be a project of historians or cultural elites; citizens also need to actively contribute on a large scale (Hirst & Manier, 2008). Recognizing this, an increasing number of museums, memorial sites, and historical archives have created opportunities for participation of the public (Antweiler, 2023; Huvila, 2008; Simon, 2010). We present causal evidence on the impact of active participation in a digital-history project. As part of this real-world, large-scale remembrance project about Nazi persecution, volunteers have digitized more than 6 million historical documents. We asked whether and how contributing to this project changed those who contributed.

Legacies of past injustice, such as slavery, genocide, colonialism, and authoritarianism, deeply divide societies and cast a long shadow on social relations today (Acharya et al., 2016; Charnysh & Finkel, 2017; Cunningham et al., 2021; Simpser et al., 2018). It is essential for citizens to confront these histories for two main reasons. First, keeping the memory of victims of past atrocities alive offers symbolic justice (Balcells & Voytas, 2023). Motivating people, especially members of the historical perpetrator groups, to engage with the injustice supports this goal. Studies that expose people to factual information about historical atrocities, using experimental laboratory or survey designs, have found that this can increase their recognition of them as an injustice and increase feelings of collective guilt (Doosje et al., 1998; Martinovic et al., 2018; Zebel et al., 2008) if they engage with the information as opposed to fending it off (Cˇehajić-Clancy et al., 2011; Gunn & Wilson, 2011; Hideg & Wilson, 2020; Leach et al., 2013; Peetz et al., 2010). Second, engaging with historical atrocities can improve intergroup relations today. Exposing people to historical injustice through critical history education (Bonam et al., 2019), museums (Balcells et al., 2022), and memorials (Turkoglu et al., 2023) has been shown to increase recognition of ongoing racial inequalities and strengthen support for democratic values, institutions, and actors today.

Despite its great potential, societies often downplay or avoid historical injustice, creating many psychological barriers to engagement with difficult pasts at the individual level (Bilewicz et al., 2017; Cohen, 2013; Hirst et al., 2018). In this context, it would be especially useful to know how people become mobilized to contribute to collective remembrance efforts and then potentially extend their efforts to improved intergroup relations today. Participatory formats may offer a particularly valuable solution. Across varied domains such as education and health, a powerful way to affect psychological change is through “performance-based procedures” (Ajzen, 1985; Bandura, 1982). When successfully performing a behavior, people develop efficacy, an important predictor of intentions for future behavior (Alessandri et al., 2009). People with high efficacy in a specific domain believe that they individually or their group can successfully reach their goals through action (Bandura et al., 2006). Once developed in one domain, efficacy beliefs can spill over to other domains that are perceived as conceptually related (Bandura, 1977; e.g., the belief that one can cope with a specific animal phobia can generalize to other animals and even fear of social situations). Participative efficacy extends this concept to individual participation in collective action (van Zomeren et al., 2013): the belief that one’s individual action matters for reaching a desirable, collective goal. We applied this idea to participatory remembrance projects and peoples’ belief that they can successfully contribute to building collective memory and that their actions matter for keeping the past alive.

Heeding calls to examine intergroup interventions in field settings (Paluck et al., 2021), we tested the causal impact of participating in a real-world, large-scale remembrance project about Nazi persecution: #everynamecounts. This innovative project offers anyone with access to a digital device a high-impact opportunity for participation: contributing to a digital-knowledge site hosted by the Arolsen Archives, the largest worldwide archive documenting national socialist (NS) persecution. The #everynamecounts project crowdsources the task of digitizing original Nazi documents, primarily from concentration camps. Once these files are digitized, descendants of victims and survivors can find out more details about the fate of their ancestors, often for the first time. In an interview with *The New York Times*, Paul Shapiro, director of international affairs for the U.S. Holocaust Memorial, said that the project’s greatest value may be to “keep the past alive” (Curry, 2020, para. 35). Thanks to the citizen-science format, anyone with 15 min to spare can make an important contribution and thus have a successful performance experience here. This invitation to contribute distinguishes #everynamecounts and other participatory projects from more passive, educational formats that primarily disseminate information (Brauer, 2024).

**Statement of Relevance**

Many memorial sites, museums, and archives are adopting participatory formats to engage people actively with historical injustices, aiming to prevent such injustices from recurring. This study examined the causal impact of a large-scale, innovative, crowd-sourced project in which volunteers have digitized more than 6 million original files documenting Nazi persecution. It revealed that participation increased people’s motivation to act collectively for further commemoration and for better intergroup relations today, particularly in countering antisemitism. Our findings highlight the potential of participatory approaches, compared with traditional methods focused on information dissemination, for mobilizing support for symbolic justice and better intergroup relations.

We predicted that active participation in remembrance projects such as #everynamecounts would mobilize people for symbolic justice regarding the past by enhancing their efficacy beliefs (Bandura, 1977; Ditlmann et al., 2017; van Zomeren et al., 2013). As noted above, efficacy beliefs can extend into related areas (Bandura, 1982). What counts as a related area may be socially constructed. Holocaust memory in museums or schools, for example, is often linked with taking responsibility for positive intergroup relations today (Antweiler, 2023). Thus, spillover might occur from commemorating the Holocaust to similar collective—and perhaps even individual—behaviors. Accordingly, we also predicted that active participation in #everynamecounts would mobilize people for better intergroup relations today. We tested these predictions with two preregistered experiments, a field-in-the-lab experiment (*n* = 552) in which we tested the impact of a real-world digital intervention compared with a theoretically motivated control condition in a laboratory setting and an online randomized control trial (RCT; *n* = 900) in which we tested the overall impact of this intervention on digital workers in a fully online setting.

## Research Transparency Statement

### General disclosures

**Conflicts of interest:** All authors declare no conflicts of interest. **Funding:** This research was supported by the Volkswagen Foundation (Volkswagen Stiftung Project No. 94937). **Artificial intelligence:** No AI-assisted technologies were used in this research or the creation of this article. **Ethics:** This research received approval from the Hertie School Research Ethics Committee (Ethics Application Approval ID 20221215-15).

### Study 1 disclosures

**Preregistration:** The hypotheses, methods, and analysis were preregistered on February 15, 2023, at https://osf.io/23ueb, and data collection started on February 16, 2023. There were minor deviations from the preregistration (for details, see Section D in the Supplemental Material available online). **Materials:** All study materials are publicly available at https://osf.io/j6gkh. **Data:** All primary data are publicly available at https://osf.io/7uma8. **Analysis scripts:** All analysis scripts are publicly available at https://osf.io/rt8zq. The file at https://osf.io/pt7ew explains how to produce the tables and figures in the main text and in the Supplemental Material. **Computational reproducibility:** The computational reproducibility of the results has been independently confirmed by the journal’s STAR team.

### Study 2 disclosures

**Preregistration:** The hypotheses, methods, and analysis were not formally preregistered but were uploaded to OSF on November 21, 2023 (https://osf.io/4u3sm) before data collection, which started on November 22, 2023. There were minor deviations from the preregistration (for details, Section D in the Supplemental Material). **Materials:** All study materials are publicly available at https://osf.io/uj8xn. **Data:** All primary data are publicly available at https://osf.io/62jau. **Analysis scripts:** All analysis scripts are publicly available at https://osf.io/e86qt. The file at https://osf.io/pt7ew explains how to produce the tables and figures in the main text and in the Supplemental Material. **Computational reproducibility:** The computational reproducibility of the results has been independently confirmed by the journal’s STAR team.

## Study 1: Field-in-the-Lab Experiment

### Method

The goal of Study 1 was to isolate the performance component of our digital-history intervention to determine whether it would have an effect that went beyond more traditional information-dissemination formats. Study 1 thus compared the effect of actively participating versus passively receiving information in the context of a digital-history project on mobilization for symbolic justice for the past, better intergroup relations today, and efficacy beliefs as well as several alternative processes. Participants first filled out a 15-min online survey containing all dependent variables.^1^ One week later, they came to a physical laboratory, at which they were randomly assigned to digital-history or information-only conditions and afterward completed all dependent variables again. After completing both parts, they were paid 20 euros. The study was run on Qualtrics.

#### Sample

We recruited student participants (i.e., participants who attended both waves) from the human subject pools of two German universities, the Experimental Laboratory for Economic Experiments at Technical University Berlin and the Mannheim Laboratory for Experimental Economics at Mannheim University.^2^ Whereas 712 students participated in the first wave, 552 participated in the second wave. According to our preregistration, to be included in our sample, participants needed to participate in both waves. Thus, our final sample size was 552, which enabled us to detect a Cohen’s *d* of 0.15 with 80% statistical power. Student participants were 55% male, and 53% were enrolled in the natural sciences or engineering. Everyone who came to the lab participated in the study. For more information on the sample, see Section A in the Supplemental Material.

#### Experimental design

**Fig. 1.**
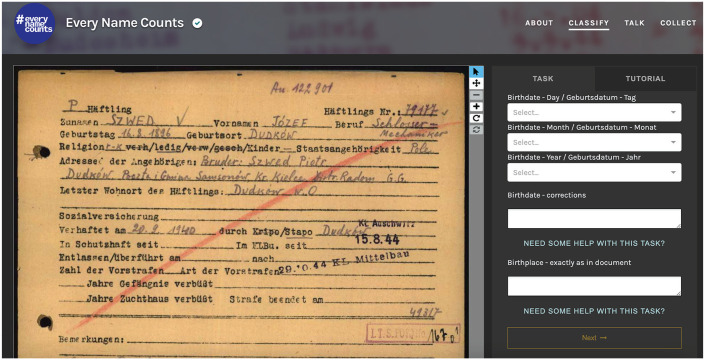
Example of digitizing the prisoner registration card of Józef Szwed for the #everynamecounts project.

Our treatment was participation in the #everynamecounts project from the Arolsen Archives, the largest archive on Nazi persecution. We informed participants that they would participate in a real-life crowdsourcing initiative and, for ethical reasons, asked them to treat the historical documents with respect (for more details on ethics, see Section C in the Supplemental Material). The treatment condition (i.e., digital-history condition) was designed to capture the typical experience people have when they contribute to the project in three parts. Participants first watched a 3-min-long instructional video adopted from the Arolsen Archives that explained the main task and its significance. Participants were then directed to the #everynamecounts project webpage and instructed to spend 15 to 20 min there while a research assistant monitored them. The platform offers an interactive interface in which participants type up information from scanned historical documents about victims into an online database. The documents were prisoner registration cards from the Buchenwald concentration camp. Many are handwritten and include names, age, and reason for “imprisonment” and sometimes family status, profession, and miscellaneous information. The interface also provides tutorials and explanations about the documents. The median value for the number of digitized documents per participant was 3 (*SD* = 1.3).^3^ An example of a prisoner registration card is shown in Figure 1. After digitizing, participants learned how many people had participated in the project until that date and how many documents had been digitized. They were then asked to complete a questionnaire with outcome questions and demographics.

To isolate the performance component of the #everynamecounts project, participants in the control condition received the same information about the archival collection and its importance without learning about or participating in the crowdsourcing project. This allowed us to separate the two main components of the #everynamecounts project: information and participation. In the control condition (i.e., information-only condition) participants read a text with information about NS persecution and the work of the Arolsen Archives. This included information about and pictures of prisoner registration cards from concentration camps—just like the ones participants in the digital-history condition digitized—and an explanation for why archiving this collection is important (for exact text in German and English, see Section B in the Supplemental Material). However, there was no reference to the #everynamecounts project, and thus participants did not participate in it. They were then asked to complete the same questionnaire with outcome questions and demographics as participants in the digital-history condition.^4^

#### Outcomes

We assessed outcomes related to symbolic justice for the past and for better intergroup relations today. For symbolic justice for the past, we measured how motivated participants were to collectively commemorate victims and how much money they suggested donating to a concentration-camp memorial site. For better intergroup relations today, we measured how motivated participants were to collectively support better intergroup relations today, how motivated they were to confront discrimination in their everyday life, and how much money they suggested donating to an antiracism foundation.^5^ To measure support for commemoration and better intergroup relations, we adopted established measures of collective action to our context (Bilali et al., 2020). For commemoration, we designed four items focused on actions of commemoration of Holocaust victims (e.g., commemorating the victims of Nazi persecution in physical space). For better intergroup relations today, we used four more typical collective-action items ensuring that each action was in support of positive intergroup relations today (e.g., participating in events that raise awareness about discrimination). To measure intentions to confront discrimination, we adopted four items based on Murrar et al. (2020) to the German context (e.g., reporting discrimination when witnessing it). All items and Cronbach’s alphas are reported in Table 1. Finally, to measure donations both for a memorial site and antiracism foundation, we used a quasi-behavioral measure. Participants could distribute 100 euros that we actually donated to the following nongovernmental organizations: KZ Gedenkstätte Dachau (concentration-camp memorial site in Germany), Amadeu Antonio Stiftung (foundation against racism and antisemitism today), Stiftung Kunstfonds (foundation for art), and the Nature and Biodiversity Conservation Union (environmental conservation organization).^6^

**Table 1.** Study 1 Outcome Items

Symbolic justice for the past
Collective action: commemoration (α = .88)
How motivated are you currently to:
– Commemorate the victims of Nazi persecution in physical space
– Commemorate the victims of Nazi persecution in digital space
– Contribute to archives about the victims of Nazi persecution
– Research your own family history in the Nazi era
Better IGR today
Collective action: IGR (α = .85)
How motivated are you currently to:
– Sign petitions against antidemocratic movements or parties
– Donate money in support of human rights work
– Join an association or initiative that takes a stand critical of group-based discrimination today
– Participate in events that raise awareness about antisemitism
Confronting discrimination (α = .88)
How motivated are you currently to:
– Say something if you see that someone is insulting a person because of their membership in a social group
– Report it to the appropriate authorities if you witness discrimination
– Reprimand someone who makes jokes about another person’s group identity
– Support the concerned person if you observe bullying on social media

Note: Participants responded to each item on a scale from 1 (*not motivated at all*) to 7 (*very motivated*). IGR = intergroup relations.

#### Processes

We assessed participative efficacy beliefs as our main proposed process (adopted from van Zomeren et al., 2013) because, as explained above, the #everynamecounts project is designed such that each participant’s contribution (digitizing the file of at least one victim) matters for reaching a group goal (digitizing the entire collection). Thus, enhanced efficacy beliefs might increase participants’ contributions to symbolic justice for the past and improved intergroup relations today.

Like any real-world intervention, #everynamecounts is complex, so it may influence people through multiple pathways. Other relevant psychological processes include perceptions of social norms and collective guilt. The mere existence of the archive and high levels of participation in the digital-history project may suggest to participants that commemorating the past (and perhaps, by extension, improving the present) is common and desirable among Germans, encouraging similar behaviors. Accordingly, we adopted five items to measure memory norms and four items to measure social norms that pertain to action for better intergroup relations today (present norms) based on Murrar et al. (2020) and Paluck (2009). Confronting information about historical atrocities in #everynamecounts may also heighten participants’ self-critical emotions, increasing their motivation for action in support of symbolic justice and better intergroup relations. Accordingly, we measured collective guilt using five items based on Branscombe et al. (2004). All items and Cronbach’s alphas are reported in Table 2.

**Table 2.** Study 1 Process Items

Participative efficacy (α = .90)
Indicate the extent to which you agree with the following statements:
– I think that I can make a meaningful contribution to helping us keep the memory of the victims of National Socialism alive.
– My contribution counts if we work together to ensure that the crimes of the National Socialists are never forgotten.
– I believe that I can make a meaningful contribution to a common future without group hatred and exclusion.
Memory norms (α = .89)
Indicate the extent to which you agree with the following statements:
– Germans often take part in initiatives for the victims of National Socialism.
– Many Germans actively contribute to the memory of the Nazi past.
– People in Germany are committed to ensuring that the victims of the Nazi regime are not forgotten.
– Many Germans find it important to keep the memory of past atrocities alive.
– For many Germans it is important to make amends with the National Socialist past.
Present norms (α = .86)
Indicate the extent to which you agree with the following statements:
– It is important to many Germans that people of other origins or religions should be treated equally.
– Many Germans dedicate time to contemporary injustice causes.
– Many Germans are active against discrimination.
– Many Germans find commitment against antisemitism a good thing.
Collective guilt (α = .78)
Indicate the extent to which you agree with the following statements:
– I feel guilty about Germans’ harmful actions toward Jews.
– I do not feel guilty about the negative things other Germans have done to Jews.^a^
– I believe I should help repair the damage caused to Jews by Germans.
– I feel regret for what Germans have done to victims of National Socialism.
– It is difficult for me to feel guilty for bad outcomes brought about by Germans.^a^

Note: Participants responded to each item on a scale from 1 (*strongly disagree*) to 7 (*strongly agree*).

a Reverse-coded.

#### Model

Following the suggestions of Clifford et al. (2021), the model of this study was defined as DV_*i*_ = β_1_ Digital History_*i*_ + β_2_ Pre-DV_*i*_ + ∈_*i*_, where DV_*i*_ refers to the dependent variables (e.g., collective-action commemoration index) for Participant *i* at Time 2 as explained above. For each dependent variable, we ran a separate model. Digital History_*i*_ refers to whether Participant *i* was in the digital-history condition, and β_1_, its coefficient, was our main interest. Participants assigned to the information-only condition were coded 0. Pre-DV_*i*_ refers to the dependent variable from the pretreatment survey (i.e., Time 1). The error term is ∈_*i*_, and robust standard errors were used. This was a between-subjects design, and each participant appeared once in the sample.

### Results

**Fig. 2.**
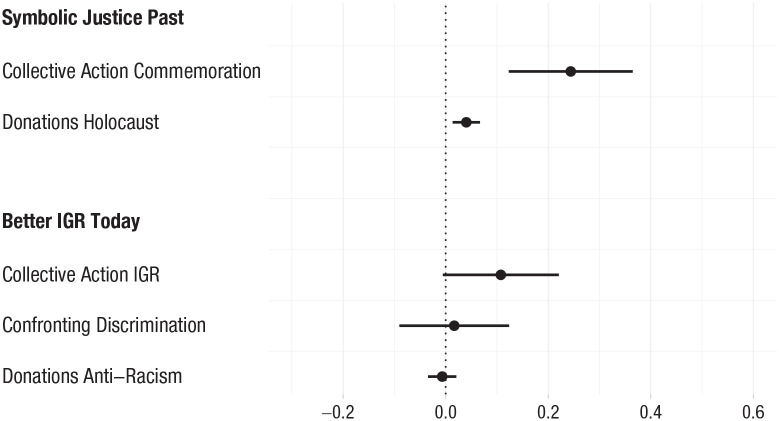
Effects of participating in #everynamecounts on mobilization compared with receiving information. Dots denote standardized coefficients for treatment and bars to 95% confidence intervals. *N* = 552. IGR = intergroup relations.

The effects of actively participating in #everynamecounts compared with merely receiving the same information are presented in Figure 2,^7^ which reports the results both for outcomes related to symbolic justice for the past and outcomes related to better intergroup relations today.^8^ Participants in the digital-history condition were more likely to engage in collective action compared with those in the information-only control condition, consistent with our predictions. The largest effect of the treatment was on collective-action intentions for commemoration (*b* = 0.24, *SE* = 0.06, *p* < .001). It also increased collective-action intentions for better intergroup relations today (*b* = 0.11, *SE* = 0.06, *p* = .06). Although this coefficient was not statistically significant at the conventional .05 level, the *p* value was .06. There was, however, no evidence that digital-history participants were more likely to confront discrimination than those in the information-only condition (*b* = 0.02, *SE* = 0.05, *p* = .76).^9^

In addition to its effects on collective-action intentions, digital-history participation also had positive effects on donation behavior. When asked to distribute 100 euros among four organizations—a concentration-camp memorial site, a foundation against racism and antisemitism, an art foundation, and an environmental conservation organization—participants in the digital-history condition on average donated 4 euros more to the concentration-camp memorial site than participants in the information-only condition (*b* = 4, *SE* = 1.4, *p* = .003).^10^ There was no significant effect on donations to the foundation against racism and antisemitism (*b* = −0.6, *SE* = 1.4, *p* = .63).^11^

**Fig. 3.**
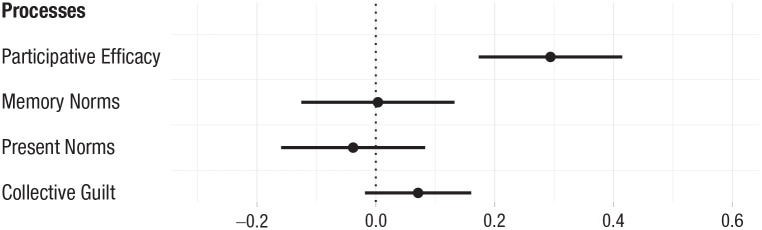
Effects of participating in #everynamecounts on processes compared with receiving information. Dots denote standardized coefficients for treatment and bars to 95% confidence intervals. *N* = 552.

The effects of participating in #everynamecounts on processes compared with receiving information only are reported in Figure 3. The results were consistent with the hypothesis that digital-history participation would affect outcomes via enhanced efficacy beliefs. Participants in the digital-history condition reported higher participative efficacy than participants in the control condition (*b* = 0.31, *SE* = 0.07, *p* < .001). Meanwhile, there were no significant differences between participation versus receiving information only on perceptions of social norms—both related to memory (*b* = 0.004, *SE* = 0.07, *p* = .96) and the present (*b* = −0.04, *SE* = 0.06, *p* = .54)—and collective guilt (*b* = 0.07, *SE* = 0.05, *p* = .12). Thus, the increases in collective-action intentions we observed in Study 1 likely resulted from increases in participants’ participative efficacy.^12^

## Study 2: Online RCT

### Method

Having established the causal role of actively participating compared with merely receiving information about the same archive and historical documents, the goal of Study 2 was to understand the overall impact of a large-scale participatory remembrance project compared with not receiving any treatment. Accordingly, Study 2 tested the overall short- and long-term impact of #everynamecounts on mobilization for symbolic justice, better intergroup relations today, and efficacy beliefs as well as several alternative processes. Digital workers were randomly assigned to the same digital-history condition as students in Study 1 or went directly to the outcome measures in a neutral, empty control condition. They filled out two surveys: the first one immediately after the treatment and the second one 2 to 3 weeks later. Participants were paid 8 euros for the first round of the study and 3 euros for the second round. The study was run on Qualtrics.

#### Sample

Because the core task in #everynamecounts is digitizing files, we recruited digital workers living in Germany for Study 2 via Clickworker, a platform for freelance digital work, and invited them to participate in a study “on attitudes toward social issues.”^13^ To measure long-term impact, we recontacted all participants 2 weeks later and invited them to a second survey. A total of 900 participants completed the first round (88% of people who clicked on the link completed the study), and 487 participants completed the second round, 2 to 3 weeks later, enabling us to detect a Cohen’s *d* of .19 and .26, respectively, with 80% statistical power. Of the 900 digital workers that enrolled in the first round, 59% were male, the average age was 40 years (range: 18–76), and all indicated support for one of the major national parties—22% CDU/CSU, 22% Greens, 13% SPD, and 10% AfD. For more information on the sample, see Section A in the Supplemental Material.

#### Experimental design

Participants were randomly assigned to participate in #everynamecounts (i.e., digital-history condition) or to go directly to the posttreatment survey (i.e., control condition). In the digital-history condition, participants watched the same instructional video as in Study 1 and then accessed the project platform hosted by our partner organization with the instruction to spend 15 to 20 min there. The median value for the number of digitized documents per participant was 3 (*SD* = 1.3). The collection of historical documents was the same as in Study 1. In the empty control condition, participants went directly to the posttreatment survey.^14^ For more information on the experimental design, including the text that participants in the control condition read, see Section B in the Supplemental Material.

#### Outcomes

As in Study 1, we assessed outcomes related to symbolic justice for the past and for a better society today. For symbolic justice for the past, we measured how mobilized participants were to commemorate victims using a refined version of the same collective-action index^15^ and how much money they suggested donating to a memorial site using the same quasi-behavioral measure as before. We added a collective-action index for commemorating colonial history (two items) to see whether spillover could occur from one historical atrocity to another. For better intergroup relations today, we measured motivation to take action in support of better intergroup relations today, motivation to confront discrimination, and donations to an antiracism foundation using the same items as before. We added a measure of collective-action intentions to combat antisemitism (four items) because exploratory analyses of the results from Study 1 suggested especially strong effects for the antisemitism item within the index for the mobilization for better intergroup relations today. All items and Cronbach’s alphas are reported in Table 3.

**Table 3.** Study 2 Outcome Items

Symbolic justice for the past
Commemoration: Holocaust (α = .93)
How motivated are you currently to:
– Commemorate the victims of Nazi persecution in physical space
– Commemorate the victims of Nazi persecution in digital space
– Contribute to archives about the victims of Nazi persecution
Commemoration: colonialism (α = .92)
How motivated are you currently to:
– Commemorate the victims of German colonialism
– Support archives that document injustice in connection with German colonial history
Better IGR today
Collective action: IGR (α = .90)
How motivated are you currently to:
– Sign petitions against antidemocratic movements or parties
– Donate money in support of human rights work
– Join an association or initiative that takes a stand critical of group-based discrimination today
– Participate in events that raise awareness about discrimination today
Collective action: antisemitism (α = .92)
How motivated are you currently to:
– Sign petitions against antisemitism
– Donate money in support of projects fighting against antisemitism
– Join an association or initiative that takes a stand critical of antisemitism today
– Participate in events that raise awareness about antisemitism
Confronting discrimination (α = .91)
How motivated are you currently to:
– Say something if you see someone insulting a person because of their membership in a social group
– Report it to the appropriate authorities if you witness discrimination
– Reprimand someone who makes jokes about another person’s group identity
– Support the concerned person if you observe bullying in social media

Note: Participants responded to each item on a scale from 1 (*not motivated at all*) to 7 (*very motivated*). IGR = intergroup relations.

We again measured efficacy beliefs as processes. Given their central role, we used refined measures by disaggregating participative, self-efficacy, and group efficacy in Study 2. We measured social norms and collective guilt as potential alternative processes as in Study 1. All items and Cronbach’s alphas are reported in Table 4.

At Time 2, we repeated the mobilization measures and added several behavioral measures: donating money as in Study 1, a chance to write a letter to the president expressing concern about vandalism at Holocaust memorial sites, and clicking on a link to learn about a different remembrance project. For more details, see Section L in the Supplemental Material.

#### Model

The model of this study was defined as DV_*i*_ = β_1_ Digital History_*i*_ + ∈_*i*_, where DV_*i*_ refers to the dependent variables (i.e., indices) for Participant *i*. For each dependent variable, a separate model was run. Digital History_*i*_ refers to whether Participant *i* was in the digital-history condition (i.e., participating in #everynamecounts), and β_1_, its coefficient, was our main interest. The error term is ∈_*i*_, and robust standard errors were used.

### Results

**Fig. 4.**
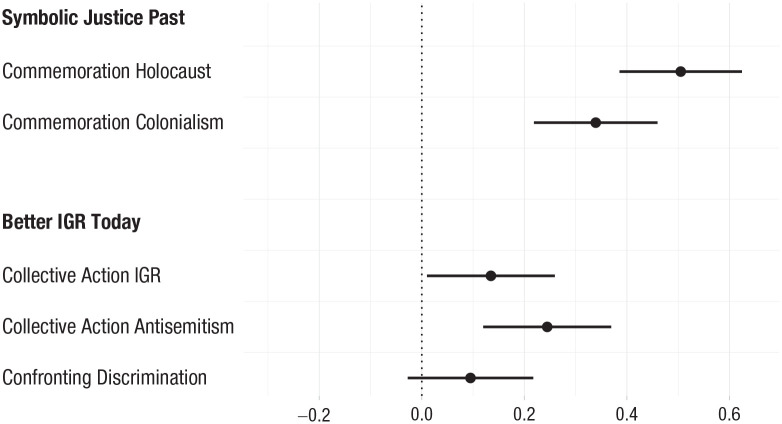
Immediate effects of participating in #everynamecounts compared with a neutral control condition. Dots denote standardized coefficients for treatment and bars to 95% confidence intervals. *N* = 900. IGR = intergroup relations.

The immediate effects of participating in #everynamecounts are presented in Figure 4,^16^ and the results corroborated the findings of Study 1. Digital-history participants were significantly more motivated to engage in collective action compared with those who did not participate. Again, the largest effect of digital-history participation, approximately 0.5 *SD*s (*SE* = 0.06, *p* < .001), was on collective-action intentions for commemoration. The effect also extended to the commemoration of victims of German colonialism by approximately 0.34 *SD*s (*SE* = 0.06, *p* < .001).^17^ As in Study 1, digital-history participation increased collective-action intentions for better intergroup relations today, but the effect, approximately 0.14 *SD*s (*SE* = 0.06, *p* = .03), was only marginally significant this time. It significantly increased collective-action intentions against antisemitism by 0.25 *SD*s (*SE* = 0.06, *p* < .001).^18^ We also preregistered an analysis of heterogeneous treatment effects by party preferences but did not find any significant differences by party (see Section I in the Supplemental Material).

**Table 4.** Study 2 Process Items

Participative efficacy (α = .88)
Indicate the extent to which you agree with the following statements:
– I believe that my contribution counts if we work together to ensure that the crimes of the National Socialists are never forgotten.
– I believe that I can personally make a meaningful contribution if we work together to shape a future without group hatred and exclusion.
Self-efficacy (α = .83)
Indicate the extent to which you agree with the following statements:
– I believe that I can personally ensure that the crimes of the National Socialists are never forgotten.
– I believe that I can personally create a future without group hatred and exclusion.
Collective efficacy (α = .84)
Indicate the extent to which you agree with the following statements:
– I believe that German society, together, can ensure that the crimes of the National Socialists are never forgotten.
– I believe that German society, together, can shape a future without group hatred and exclusion.
Memory norms (α = .91)
Indicate the extent to which you agree with the following statements:
– Germans often take part in initiatives for the victims of National Socialism.
– Many Germans actively contribute to the memory of the Nazi past.
– People in Germany are committed to ensuring that the victims of the Nazi regime are not forgotten.
– Many Germans find it important to keep the memory of past atrocities alive.
– For many Germans it is important to make amends with the National Socialist past.
Present norms (α = .88)
Indicate the extent to which you agree with the following statements:
– It is important to many Germans that people of other origins or religions should be treated equally.
– Many Germans dedicate time to contemporary injustice causes.
– Many Germans are active against discrimination.
– Many Germans find commitment against antisemitism a good thing.
Collective guilt (α = .81)
Indicate the extent to which you agree with the following statements:
– I feel guilty about Germans’ harmful actions toward Jews.
– I do not feel guilty about the negative things other Germans have done to Jews.^a^
– I believe I should help repair the damage caused to Jews by Germans.
– I feel regret for what Germans have done to victims of National Socialism.
– It is difficult for me to feel guilty for bad outcomes brought about by Germans.^a^

Note: Participants responded to each item on a scale from 1 (*strongly disagree*) to 7 (*strongly agree*). ^a^Reverse-coded.

**Fig. 5.**
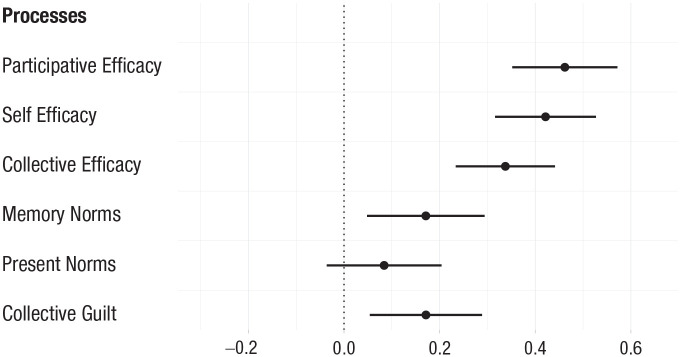
Immediate effects of participating in #everynamecounts on processes compared with a neutral control condition. Dots denote standardized coefficients for treatment and bars to 95% confidence intervals. *N* = 900.

An analysis of possible processes confirmed the important role of efficacy beliefs as already observed in Study 1. The immediate effects of participating in #everynamecounts on processes are reported in Figure 5. Contributing to digital history significantly increased all three types of efficacy. The effects were larger for participative efficacy (*b* = 0.46, *SE* = 0.06, *p* < .001) and self-efficacy (*b* = 0.42, *SE* = 0.05, *p* < .001) compared with group efficacy (*b* = 0.34, *SE* = 0.05, *p* < .001).^19^ Unlike Study 1, compared with a neutral control condition in Study 2, #everynamecounts also increased collective guilt (*b* = 0.17, *SE* = 0.06, *p* = .004) and perceived memory norms (*b* = 0.17, *SE* = 0.06, *p* = .006) but not perceived social norms about taking action for better intergroup relations today (*b* = 0.08, *SE* = 0.06, *p* = .17).

**Fig. 6.**
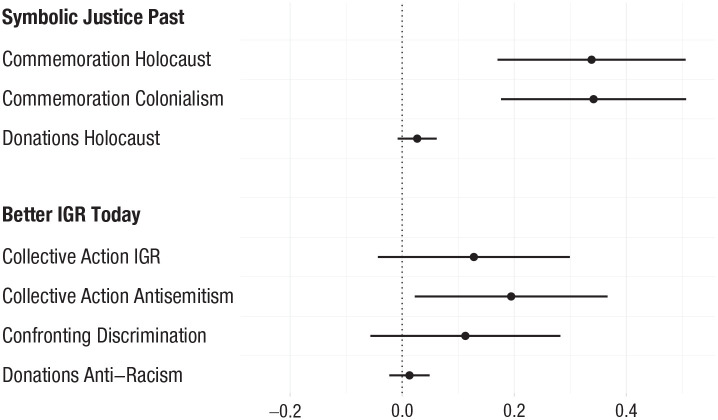
Effects of participating in #everynamecounts 2 to 3 weeks after the intervention compared with a neutral control condition. Dots denote standardized coefficients for treatment and bars to 95% confidence intervals. *N* = 487. IGR = intergroup relations.

The long-term effects of participating in #everynamecounts 2 to 3 weeks later are presented in Figure 6. Compared with the immediate effects, a decreased yet still substantive effect was observed on intentions to commemorate victims of NS persecution (*b* = 0.34, *SE* = 0.09, *p* < .001). The effect on commemorating the victims of colonialism persisted at approximately 0.34 SDs (*SE* = 0.08, *p* < .001). We also continued to observe a positive effect on mobilization against antisemitism at approximately 0.19 SDs (*SE* = 0.09, *p* = .03). Although we did not observe significant effects on action intentions for better intergroup relations more generally, the effect sizes in both surveys were similar (0.135 *SD*s in the first and 0.128 *SD*s in the second).

Study 1 showed that participating in a remembrance project had immediate positive effects on donation behavior (see Fig. 2). Although we observed increases in donations to a concentration-camp memorial site by approximately 2.7 euros (*SE* = 1.7, *p* = .13) 2 to 3 weeks after the intervention in Study 2, this was not statistically significant.^20^ We also found no direct effects of digital-history participation on writing a letter to the president or clicking on a link about a different remembrance project 2 to 3 weeks later.

There are two main possible ways to interpret these behavioral outcomes findings. First, the effects on behaviors abated over time. Although we observed immediate significant effects in Study 1, we did not observe any significant effects in 2 to 3 weeks in Study 2. A possible justification for this interpretation is that we observed a decrease in commemoration intentions between the first and second surveys in Study 2, and, in a similar vein, the effect of treatment on behaviors would have abated over time. The decrease in commemoration intentions was around 33% (from .51 to .34). Assuming the same decrease, we would have expected a donation of 3.6 euros in the first wave, which was close to the effect size in Study 1 (increase in donation of 4 euros), and our study was well powered to detect such an effect size. A second, alternative interpretation is of course that the digital-history intervention did not have significant effects on any of the behaviors we measured.

## Summary of Findings

This section offers a summary of the main findings. Table 5 offers the list of hypotheses as preregistered; Table 6 provides information for each hypothesis on what measures were used, whether there were significant effects, and whether the effects were in the hypothesized direction for Study 1; and Table 7 provides the same information for Study 2.

**Table 5.** Hypotheses

Hypothesis 1a: Participating in memory work mobilizes participants to engage in further memory work compared with only receiving information about historic injustice.
Hypothesis 1b: Participating in memory work mobilizes participants to act against intergroup injustice today compared with only receiving information about historic injustice.
Hypothesis 2a: Participating in memory work increases participants’ perception that Germans contribute to memory work compared with only receiving information about historic injustice.
Hypothesis 2b: Participating in memory work increases participants’ perception that Germans act against intergroup injustice today compared with merely receiving information about historic injustice.
Hypothesis 3: Participating in memory work improves participants’ beliefs about societal out-groups compared with only receiving information about historic injustice.

**Table 6.** Study 1 Findings

Hypothesis	Outcome	Effect direction	Direction anticipated?	Effect significant?	Reference
1a	Collective action—commemoration	Positive	Yes	Yes	Fig. 2
1a	Donations—Holocaust	Positive	Yes	Yes	Fig. 2
1b	Collective action—IGR	Positive	Yes	No	Fig. 2
1b	Confronting discrimination	Positive	Yes	No	Fig. 2
1a	Donations—antiracism	Negative	No	No	Fig. 2
2a	Memory norms	Positive	Yes	No	Fig. 3
2b	Present norms	Negative	No	No	Fig. 3
3	Rejecting political correctness	Positive	Yes	No	Table S4
3	Feeling thermometer	Positive	Yes	No	Table S4
3	Colorblindness	Negative	No	No	Table S4

Note: IGR = intergroup relations.

**Table 7.** Study 2 Findings

Hypothesis	Wave	Outcome	Effect direction	Direction anticipated?	Effect significant?	Reference
1a	1	Commemoration—Holocaust	Positive	Yes	Yes	Fig. 4
1a	2	Commemoration—Holocaust	Positive	Yes	Yes	Fig. 6
1a	1	Commemoration—colonialism	Positive	Yes	Yes	Fig. 4
1a	2	Commemoration—colonialism	Positive	Yes	Yes	Fig. 6
1a	2	Donations—Holocaust	Positive	Yes	No	Fig. 6
1a	2	Writing a letter	Positive	Yes	No	Table S12
1a	2	Interest in Stolpersteine	Positive	Yes	No	Table S12
1b	1	Collective action—antisemitism	Positive	Yes	Yes	Fig. 4
1b	2	Collective action—antisemitism	Positive	Yes	Yes	Fig. 6
1b	1	Collective action—IGR	Positive	Yes	Yes	Fig. 4
1b	2	Collective action—IGR	Positive	Yes	No	Fig. 6
1b	1	Confronting discrimination	Positive	Yes	No	Fig. 4
1b	2	Confronting discrimination	Positive	Yes	No	Fig. 6
1a	2	Donations—antiracism	Positive	Yes	No	Fig. 6
2a	1	Memory norms	Positive	Yes	Yes	Fig. 5
2b	1	Present norms	Positive	Yes	No	Fig. 5
3	1	Feeling thermometer	Positive	Yes	No	Table S6
3	1	Colorblindness	Positive	Yes	No	Table S6

## Discussion

Two studies, one field-in-the-lab experiment and one online RCT, provided causal evidence that participating in a real-world large-scale remembrance project mobilizes people to engage in further commemoration activities and to support collective action for better intergroup relations today. Across the two studies, participation strongly enhanced participative efficacy beliefs, which likely drive these mobilization effects. Compared with a neutral control condition, the intervention also significantly increased perceived social norms and collective guilt (Study 2). However, when isolating the effect of participation compared with receiving the same information without the opportunity to participate, the intervention increased only participative efficacy (Study 1). Together, these findings suggest that enhanced efficacy beliefs are the process that distinguishes participatory memory work from other, more passive formats of learning about past atrocities and injustice. By integrating work on symbolic transitional justice with research on behavior change via efficacy beliefs (Bandura, 1977), the current research thus showcases what a behavioral perspective can add to research on collective memory.

Notably, the observed mobilization effects extended to the commemoration of another instance of past injustice: colonialism. This finding provides evidence for the potential of multilateral memory (Rothberg, 2009), which stands in contrast to zero-sum thinking. Rather than a competition for attention, our results point to a virtuous cycle of commemorating one instance of past injustice for commemorating others, resonating with research on inclusive victimhood (Vollhardt & Bilali, 2015).

We also observed weaker but statistically significant effects on mobilization for collective action on improved intergroup relations today, especially antisemitism. Efficacy beliefs and performance experiences mutually reinforce each other over time (Bandura, 1977). Each time citizens successfully contribute to digital history, this strengthens their efficacy beliefs, which in turn should motivate them to engage in further action. This highlights how important #everynamecounts and similar participatory projects can be as catalysts for action in what is sometimes referred to as “dynamic systems” (Cikara et al., 2022). Interestingly, although this is true for outcomes that tap into collective processes, we found no evidence for the more individualized intention to confront discrimination in interpersonal encounters. Such outcomes might be more responsive to role modeling of individual actions (e.g., moral exemplars; Witkowska et al., 2019).

One limitation of our study is the nature of the samples. Ideally, we wanted to study the project as it rolled out naturally, but it was impossible for ethical and practical reasons to randomize. Instead, we selected study participants and settings similar to the ones in which digital-history projects typically take place: educational institutions and the workplace. An advantage of this— less naturalistic—strategy is that we minimized selection. Additionally, our samples were university students and digital workers in Germany, the latter covering broad segments of society (for sample characteristics, see Table S2 in the Supplemental Material). Further research is required to understand the generalizability of our findings to other countries and other historical atrocities.

By examining the impact of a particularly innovative, real-world project, we have presented the first-ever causal evidence on the potential impact participatory digital-history projects can have compared with more common formats that educate via information dissemination. The United Nations considers Holocaust education central for promoting global citizenship (Sustainable Development Goal 4.7) and has called on all member states to “develop educational programmes that will inculcate future generations with the lessons of the Holocaust in order to help prevent future genocides” (UNESCO, 2017, p. 15). But empirical evidence on the effect of Holocaust education on changing attitudes and behaviors is scarce (Stevick, 2018). Our study demonstrates that participatory formats can mobilize people by strengthening their beliefs that they can meaningfully contribute to collective remembrance. Our results for decreasing prejudice, discussed in Section E in the Supplemental Material, are less optimistic, echoing results from other large-scale intergroup interventions that have found positive results for behavioral intentions but not for intergroup attitudes (Mousa, 2020; Paluck et al., 2021; Scacco & Warren, 2018).

An increasing number of memorial sites, museums, and historical archives around the world are creating opportunities for public participation. Keeping in mind additional challenges that come from engagement with conflict archives (Luft, 2020; Skarpelis, 2020; for details, see Section C in the Supplemental Material), our research demonstrates the potential of participatory approaches for mobilizing people in support of symbolic justice and better intergroup relations. This kind of active engagement in collective memory work may thus have positive downstream effects for strengthening liberal, pluralistic democracies more broadly.

## Supplemental Material

sj-pdf-1-pss-10.1177_09567976251331040 – Supplemental material for Participating in a Digital-History Project Mobilizes People for Symbolic Justice and Better Intergroup Relations Today Supplemental material, sj-pdf-1-pss-10.1177_09567976251331040 for Participating in a Digital-History Project Mobilizes People for Symbolic Justice and Better Intergroup Relations Today by Ruth Ditlmann, Berenike Firestone and Oguzhan Turkoglu in Psychological Science

## Transparency

*Action Editor:* Roni Porat

*Editor:* Simine Vazire

*Author Contributions*

**Ruth Ditlmann:** Conceptualization; Data curation; Funding acquisition; Methodology; Writing – original draft; Writing – review & editing.

**Berenike Firestone:** Conceptualization; Data curation; Methodology; Writing – original draft; Writing – review & editing.

**Oguzhan Turkoglu:** Conceptualization; Data curation; Formal analysis; Methodology; Writing – original draft; Writing – review & editing.

**Correction (September 2025):** This article has been updated with edits to Cronbach’s alphas in Table 3.

**Artifical Intelligence:** No AI-assisted technologies were used in this research or the creation of this article.

**Ethics:** This research received approval from the Hertie School Research Ethics Committee (Ethics Application Approval ID 20221215-15).

**Open Practices:** Study 1 disclosures. Preregistration: The hypotheses, methods, and analysis were preregistered on February 15, 2023, at https://osf.io/23ueb, and data collection started on February 16, 2023. There were minor deviations from the preregistration (for details, see Section D in the Supplemental Material available online). Materials: All study materials are publicly available at https://osf.io/j6gkh. Data: All primary data are publicly available at https://osf.io/7uma8. Analysis scripts: All analysis scripts are publicly available at https://osf.io/rt8zq. The file at https://osf.io/pt7ew explains how to produce the tables and figures in the main text and in the Supplemental Material. Computational reproducibility: The computational reproducibility of the results has been independently confirmed by the journal’s STAR team. Study 2 disclosures. Preregistration: The hypotheses, methods, and analysis were not formally preregistered but were uploaded to OSF on November 21, 2023 (https://osf.io/4u3sm) before data collection, which started on November 22, 2023. There were minor deviations from the preregistration (for details, Section D in the Supplemental Material). Materials: All study materials are publicly available at https://osf.io/uj8xn. Data: All primary data are publicly available at https://osf.io/62jau. Analysis scripts: All analysis scripts are publicly available at https://osf.io/e86qt. The file at https://osf.io/pt7ew explains how to produce the tables and figures in the main text and in the Supplemental Material. Computational reproducibility: The computational reproducibility of the results has been independently confirmed by the journal’s STAR team.

## Notes

1. Participants filled out the outcomes questionnaire 1 week before the experiment so that we would be able to control for pre-experiment scores in the analysis to increase precision (Clifford et al., 2021).

2. At Mannheim University, participants were recruited on February 27–28, 2023. At Technical University Berlin, participants were recruited on February 23–24, March 28–29, and April 18, 2023.

3. More information about the documents is reported in Section B in the Supplemental Material.

4. To complete the task and fill out the questionnaire, participants were scheduled for 45-min laboratory sessions in both conditions.

5. We also measured prejudice through three indices—feeling thermometer, colorblindness, and defensiveness against political correctness—and found no effects. For brevity, prejudice was not included in the main text. For theory, measurement, and results on prejudice, see Section E in the Supplemental Material.

6. Participants answered the outcome questions twice (pre- and posttreatment). Answering questions about commemoration and intergroup relations in the first round might have affected their answers in the second round. To minimize possible testing effects, we included distraction questions about their intentions to live a healthy life, socialize with friends, and Schwartz values (Schwartz, 2012) in the first round.

7. All analyses presented here were preregistered. For further information on the preanalysis plan, see Section D in the Supplemental Material.

8. In between data collection and analysis, we realized that about half of the sample consisted of individuals with a migration background (i.e., at least one parent was born outside of Germany). Given the lack of intergenerational ties to the Nazi past, we preregistered that treatment effects would likely be smaller for participants with a migration background than for participants without a migration background. However, there were no significant effects in support of our hypothesis, and we thus do not report these results. For more information, please see Section D in the Supplemental Material.

9. We also report the boxplots of all outcomes by condition in Section G in the Supplemental Material.

10. To present all results in the same graph, we plotted the effects of treatment on donation in proportion rather than the actual value (i.e., 0.04) in Figure 2. Values for other measures were standardized by standard deviation.

11. Although there was no effect on donations to Stiftung Kunstfonds (foundation for art; *b* = 0.01, *SE* = 0.1, *p* = .58), there was a significant negative effect on donations to the Nature and Biodiversity Conservation Union (environmental conservation organization; *b* = −0.04, *SE* = 0.02, *p* = .02). Because participants allocated 100 euros among organizations, it seems that the increase in donations to the memorial site mostly came at the expense of donations to the environmental organization.

12. We also report the correlations between participative efficacy and our outcomes in the Table S8 in the Supplemental Material.

13. Considering the intensity of our treatment for an online study, we used an attention-check question. In the middle of the survey, we asked participants in both conditions to select “red” to continue the survey with the options of red, blue, yellow, and green. In total, four participants failed the attention check and were excluded from the analyses.

14. The median time spent on the overall survey was approximately 36.8 min in the digital-history condition (including digitizing the files), whereas it was 8.9 min in the control condition. In the second survey, 2 to 3 weeks later, the median time spent on the survey was 5.4 min.

15. In Study 1, we measured action intentions for commemoration with four items. Although three focused on the country’s history, one item focused on individual family history. A detailed analysis suggested that participating in #everynamecounts did not have significant effects on the item about individual family history.

16. All the analyses presented here were preregistered. For further information on the preanalysis plan, see Section D in the Supplemental Material.

17. For a discussion on selective attrition and the robustness of the results, see Section J in the Supplemental Material.

18. We also report the boxplots of all outcomes by condition in Section G in the Supplemental Material.

19. We also report the correlations between the efficacy indices and our outcomes in Table S9 in the Supplemental Material.

20. In Study 2, we were interested in the effects of participating in #everynamecounts 2 to 3 weeks after the intervention. We could not explore immediate effects because the treatment was related to taking action, and any behavioral outcome provides an avenue to act. Thus, behavioral measures in the first survey would have contaminated the control condition and may have prevented us from examining the effects of treatment 2 to 3 weeks after the intervention.
