# Supplementary material for: Participating in a Digital-History Project Mobilizes People for Symbolic Justice and Better Intergroup Relations Today
Source: Psychol Sci. 2025 Apr 17;36(4):249–64. doi: 10.1177/09567976251331040 (PMC13428924; doi:10.1177/09567976251331040)
Supplement: sj-pdf-1-pss-10.1177_09567976251331040 – Supplemental material for Participating in a Digital-History Project Mobilizes People for Symbolic Justice and Better Intergroup Relations Today [file sj-pdf-1-pss-10.1177_09567976251331040.pdf]

Participating in a digital history project mobilizes people for symbolic justice and better intergroup relations today

SUPPLEMENTAL MATERIALS

Contents

Section A: Sample Summary Statistics **3**

Section B: Experimental Conditions **6**

Section C: Ethics **11**

Section D: Pre-registration **12**

Section E: Effects on prejudice **15**

Section F: Within-subject effects in Study 1 **19**

Section G: Distribution of outcomes by condition **21**

Section H: Relations between outcomes and processes **24**

Section I: Heterogeneous treatment effects by party preferences in Study 2 **25**

Section J: Selective attrition in Study 2 **28**

Section K: Limiting the sample to those who attended both surveys in Study 2 **31**

Section L: Behavioral outcomes in Study 2 **32**

Section A: Sample Summary Statistics

Study 1

Table S1 reports the summary statistics for the sample in Study 1. We use binary variables for age categories, male, immigrant, German citizen, identifying as a member of victim groups of Nazi persecution, identifying as a member of a group currently discriminated against in Germany, religion categories, and a continuous variable for political ideology (0-left and 10-right). While the first column reports the averages for the full sample, the second column and third columns report the averages for participants in the control and treatment conditions, respectively. The final column reports the differences between treatment and control conditions obtained via bivariate analyses. The standard deviation for the first three columns and standard errors for the final column are in parentheses. As can be seen in the table, randomization of the treatment worked well and there are no significant differences between conditions regarding demographics.

Study 2

Table S2 reports the summary statistics for the sample in Study 2. We use binary variables for male, university degree, immigrant, German citizen, living in East Germany, living in Berlin, living in West Germany, identifying as a member of victim groups of Nazi persecution, identifying as a member of a group discriminated against in Germany, and religion categories, and continuous variables for age and political ideology (0-left and 10-right). While the first column reports the averages for the full sample, the second column and third columns report the averages for participants in the control and treatment conditions respectively. The final column reports the differences between treatment and control conditions obtained via bivariate analyses. The standard deviation for the first three columns and standard errors for the final column are in parentheses. The sample is more diverse than in Study 1. It has young (e.g., 18 years old) and old participants (e.g., 76 years old), highly educated and less educated participants, and participants from different parts of Germany (covering both East and West Germany). As can be seen in the table, randomization of the treatment worked well and there are no significant differences between conditions regarding demographics.

Table S1: Summary Statistics of Participants in Study 1

Outcome	Full sample	Control	Treatment	Difference
Age: 18-20	0.114	0.113	0.115	0.002
	(0.318)	(0.317)	(0.32)	(0.027)
Age: 21-25	0.473	0.485	0.460	-0.025
	(0.500)	(0.501)	(0.499)	(0.043)
Age: 26-30	0.275	0.259	0.291	0.032
	(0.447)	(0.439)	(0.455)	(0.038)
Age: 30+	0.074	0.073	0.076	0.003
	(0.262)	(0.261)	(0.265)	(0.022)
Male	0.566	0.551	0.581	0.030
	(0.496)	(0.498)	(0.494)	(0.043)
Immigrant	0.489	0.511	0.468	-0.043
	(0.500)	(0.501)	(0.500)	(0.043)
German citizen	0.819	0.818	0.820	0.003
	(0.385)	(0.387)	(0.385)	(0.033)
Identify as victim	0.188	0.208	0.169	-0.039
	(0.391)	(0.407)	(0.375)	(0.033)
Identify as discriminated	0.317	0.321	0.313	-0.008
	(0.466)	(0.468)	(0.465)	(0.040)
Political ideology	3.486	3.464	3.509	0.046
	(1.849)	(1.86)	(1.841)	(0.158)
Religion: Christian	0.299	0.292	0.307	0.015
	(0.458)	(0.456)	(0.462)	(0.039)
Religion: Atheist	0.539	0.544	0.534	-0.009
	(0.499)	(0.499)	(0.500)	(0.043)
Religion: Others	0.161	0.164	0.158	-0.006
	(0.368)	(0.371)	(0.366)	(0.031)

The first column reports the average for the full sample, the second column for the control condition, and the third column for the treatment condition. The final column reports the difference between treatment and control conditions obtained by bivariate analyses. The standard deviation for the first three columns and standard errors for the final column are in parentheses.

Table S2: Summary Statistics of Participants in Study 2

Outcome	Full sample	Control	Treatment	Difference
Age	39.686	39.99	39.322	-0.668
	(12.474)	(12.466)	(12.489)	(0.835)
Male	0.598	0.582	0.617	0.034
	(0.491)	(0.494)	(0.487)	(0.033)
University education	0.466	0.453	0.480	0.027
	(0.499)	(0.498)	(0.500)	(0.033)
Immigrant	0.183	0.173	0.195	0.022
	(0.387)	(0.379)	(0.397)	(0.026)
German citizen	0.962	0.959	0.966	0.007
	(0.191)	(0.198)	(0.182)	(0.013)
Living in East	0.157	0.153	0.161	0.008
	(0.364)	(0.360)	(0.368)	(0.024)
Living in Berlin	0.081	0.082	0.080	-0.001
	(0.273)	(0.274)	(0.272)	(0.018)
Living in West	0.762	0.765	0.759	-0.007
	(0.426)	(0.424)	(0.428)	(0.029)
Identify as victim	0.124	0.110	0.141	0.031
	(0.33)	(0.313)	(0.349)	(0.022)
Identify as discriminated	0.193	0.208	0.176	-0.033
	(0.395)	(0.406)	(0.381)	(0.026)
Political ideology	4.513	4.500	4.529	0.029
	(1.788)	(1.845)	(1.719)	(0.119)
Religion: Christian	0.480	0.482	0.478	-0.004
	(0.500)	(0.500)	(0.500)	(0.033)
Religion: Atheist	0.447	0.443	0.451	0.008
	(0.497)	(0.497)	(0.498)	(0.033)
Religion: Others	0.073	0.076	0.071	-0.005
	(0.261)	(0.264)	(0.257)	(0.017)

The first column reports the average for the full sample, the second column for the control condition, and the third column for the treatment condition. The final column reports the difference between treatment and control conditions obtained by bivariate analyses. The standard deviation for the first three columns and standard errors for the final column are in parentheses.

Section B: Experimental Conditions

Treatment - Digital history participation: Studies 1 and 2

Our treatment in both studies was participation in the #everynamecounts project from the Arolsen Archives, the largest archive on Nazi persecution.[1] The main purpose of the project is to make their archival collections of documents on concentration camp prisoners, forced laborers under the National Socialist regime, and displaced persons available online. This online archive is open to anyone, including the descendants of victims who can use it to find out more about the fate of their ancestors. For both of our studies, participants in the digital history condition digitized documents from a concentration camp collection (Buchenwald). After being assigned to the treatment condition, participants read the following text:

> This experiment is perhaps a little unusual. We are asking you to take part in a crowdsourcing initiative. The initiative is called #everynamecounts and is led by the Arolsen Archives. As part of the initiative, volunteers are building a digital memorial to the victims of National Socialist persecution. The current study serves as scientific support for the project. You will learn more in a moment. Please remember, even though you are taking part in an experiment, this is a real-life initiative and the documents you are about to see are original historical documents containing information about the victims of National Socialism. Please treat them with care and respect.

The historical documents participants in our study digitized are so-called prisoner registration cards. The SS registered every person who was held captive in concentration camps via a prisoner registration card. The importance of these cards lies in documenting the atrocities and preserving knowledge about them for future generations. They are also often the only information about the fate of victims available to family members and their descendants. The cards vary but most include a fair amount of biographical information (e.g., birth date, occupation, and family status) and information related to Nazi persecution (e.g., prisoner number, group category assigned by the Nazis). During the time of the study, the collection that was being digitized was from the concentration camp Buchenwald, where over 250K people were imprisoned. Around one-third of the prisoners were Jews. The majority of prisoners at Buchenwald were Eastern Europeans (Poles, Russians, Czechs) who were taken captive once WWII began. Significantly smaller persecuted groups at Buchenwald included Sinti and Roma, political opponents, homosexuals, people with a criminal record, and people with disabilities.[2] Prisoner registration cards from the collection were presented at random on the screen of the #everynamecounts project.

[1] When we carried out the first study, the project was located on a citizen-science platform called Zooniverse. While there are minor differences in the interface, the content and activities are the same on the old and new project websites.

[2] For more information about the Buchenwald concentration camp and its history, please see `https://www.buchenwald.de/en/geschichte/chronologie/konzentrationslager`.

Control - Information-only condition: Study 1

Participants in the information-only control condition of Study 1 read a text about the Arolsen Archives and prisoner registration cards, and saw two example documents. These were scans of two prisoner registration cards, one of a Jewish victim and one of a Russian victim. We selected these documents to be representative of the collection. Please see the exact text (English translation and German original) as well as the scans below. Because reading the text took less time than the 15-20 minutes that participants in the digital history condition spent digitizing, participants in the control condition read an unrelated text about technology communication and answered questions about it after completing the main study. This ensured that the duration of the experiment was similar for all participants, so that everyone would leave at a similar time since multiple participants worked in separate cubicles but within the same laboratory room within sessions.

Information-only condition text translated into English: In the first part of the experiment, you will learn about historical documents from the time of National Socialist persecution. Please read the text carefully. We will then ask you a few questions.

Between 1933 and 1945, millions of people were deported and murdered under National Socialist rule. Documents about the fate of many of these people have been archived in Bad Arolsen in Hesse. Today, the documents in the Bad Arolsen archives are important for family members of the victims who want to find out more about their relatives, as well as for historians. And they are important historical evidence that makes it impossible to deny that the Nazi crimes took place.

In the following, we would like to explain the existence of these archives and their historical context in more detail. There are various documents, with prisoner registration cards and registration forms being the most common documents. These documents usually contain information about the name, date of registration, date of birth, prisoner category according to the logic of the Nazi regime, a prisoner number, family members, their address, occupation and, for some prisoners, the date of death. In addition, some cards contain further details such as information about the prisoner's appearance (e.g. height), state of health and any previous imprisonment. Sometimes the information is handwritten, sometimes typewritten, and often the handwriting is difficult to read.

The prisoner registration cards were created for the prisoners of the concentration camps in all large camps. The prisoner employees had to fill them out either by hand or using a typewriter. Sometimes a photo of the prisoner taken in the camp was glued onto the card. Each time a new prisoner arrived at a concentration camp, a prisoner registration card was created, on which all important personal details were noted.

Some prisoners had to work for the camp administration and create registration forms for the new prisoners of a concentration camp. This was one of the most important documents for managing information about prisoners in a concentration camp. When a person arrived at a concentration camp, all information about them relevant to the SS was recorded on this form: their personal details, previous imprisonments and the reasons for them, as well as sentences and transfers to other camps.

Today, this historical evidence, which makes it impossible to deny that the Nazi crimes took place, is kept in Bad Arolsen.

Below you can see some examples of what these documents looked like. Please remember,

even if you are taking part in an experiment, these are original historical documents with information about the victims of National Socialism. Please treat them with care and respect.

Information-only condition text in German (original): Im ersten Teil des Experiments lernen Sie etwas über historische Dokumente aus der Zeit der nationalsozialistischen Verfolgung. Bitte lesen Sie sich den Text dazu gut durch. Danach werden wir Ihnen einige Fragen stellen.

Zwischen 1933 und 1945 wurden unter der nationalsozialistischen Herrschaft Millionen von Menschen verschleppt und ermordet. Dokumente über das Schicksal vieler dieser Menschen sind im hessischen Bad Arolsen archiviert worden. Heute sind die Dokumente in den Bad Arolsen Archiven wichtig für Familienangehörige der Opfer, die etwas über ihre Verwandten herausfinden wollen, sowie für Historiker. Und sie sind wichtige historische Beweise, die es unmöglich machen zu leugnen, dass die NS-Verbrechen stattgefunden haben.

Im folgenden möchten wir Ihnen den Bestand dieser Archive und ihren historischen Kontext noch etwas genauer erklären. Es gibt verschiedene Dokumente, wobei Häftlingsregistrierungskarten und Registrierungsformulare die gängigsten Dokumente sind. Diese Dokumente enthalten in der Regel Informationen über den Namen, das Anmeldedatum, das Geburtsdatum, die Häftlingskategorie gemäß der Logik des NS-Regimes, eine Häftlingsnummer, Familienangehörige, ihre Adresse, ihren Beruf und bei einigen Häftlingen das Todesdatum. Darüber hinaus enthalten einige Karten weitere Angaben wie Informationen über das Aussehen des Häftlings (z.B. Größe), den Gesundheitszustand und gegebenenfalls frühere Inhaftierungen. Manchmal sind die Informationen handschriftlich, manchmal maschinenschriftlich verfasst, und oft ist die Handschrift schwer zu lesen.

Die Häftlingsregistrierungskarten wurden für die Häftlinge der Konzentrationslager in allen großen Lagern erstellt. Die Häftlingsangestellten mussten sie entweder von Hand oder mit einer Schreibmaschine ausfüllen. Manchmal wurde ein Foto des Häftlings, das im Lager aufgenommen wurde, auf die Karte geklebt. Bei jeder Ankunft eines neuen Häftlings in einem Konzentrationslager wurde eine Häftlingsregistrierungskarte erstellt, auf der alle wichtigen persönlichen Angaben vermerkt waren.

Einige Häftlinge mussten für die Lagerleitung arbeiten und für die neuen Häftlinge eines Konzentrationslagers Registrierungsformulare erstellen. Dies war eines der wichtigsten Dokumente, um die Informationen über die Häftlinge in einem Konzentrationslager zu verwalten. Wenn eine Person in ein Konzentrationslager kam, wurden alle für die SS relevanten Informationen über sie auf diesem Formular festgehalten: ihre persönlichen Daten, frühere Inhaftierungen und deren Gründe sowie Strafen und Verlegungen in andere Lager.

Heute werden diese historischen Beweise, die es unmöglich machen zu leugnen, dass die NS-Verbrechen stattgefunden haben, in Bad Arolsen aufbewahrt.

Unten sehen Sie einige Beispiele dafür, wie diese Dokumente aussahen. Denken Sie bitte daran, auch wenn Sie gerade bei einem Experiment mitmachen, sind dies historische Originaldokumente mit Informationen über die Opfer des Nationalsozialismus. Bitte gehen Sie achtsam und respektvoll damit um.

Jude, Ung

Konzentrationslager Art der Haft: Gef. Nr.: 56919

Name und Vorname: Weisz József

geb.: 3.8.1911 zu: Trebusa, kom. Marmaros

Wohnort: ~~Trebusa~~ Felsö Visso Kurhaus 43, Marmaros

Beruf: Beamter Rel.:

Staatsangehörigkeit: Ung. Stand: verheir.

Name der Eltern: Vater Fleischer Faivel W. gest. 1937 Rasse:

Wohnort: d. Mutt. Mutter Ethel W. geb. Rothenberg Trebusa, Marmaros

Name der Ehefrau: Frimet W. geb. Schwalb Rasse:

Wohnort: Felsö Visso wo.

Kinder: 3: 8, 6 J., 5 M. Alleiniger Ernährer der Familie oder der Eltern:

Vorbildung: Volksschule 4 J.

Militärdienstzeit: — von — bis

Kriegsdienstzeit: — von — bis

Grösse: 163 Gestalt: mittelstark Gesicht: oval Augen: braun

Nase: gerade Mund: klein Ohren: abstеh. Zähne: 4 fehl.

Haare: schwarz Sprache: ungarisch, deutsch

Ansteckende Krankheit oder Gebrechen: —

Besondere Kennzeichen: —

Rentenempfänger:

Verhaftet am: 21.5.44 wo: Felsö Visso

1. Mal eingeliefert: 24.5.44 Auschw. 2. Mal eingeliefert: 2.6.44 Bu.

2.6.44
KL Auschwitz

Einweisende Dienststelle:

Grund:

Parteizugehörigkeit: — von — bis

Welche Funktionen: —

Mitglied v. Unterorganisationen: —

Kriminelle Vorstrafen: —

Politische Vorstrafen: —

Ich bin darauf hingewiesen worden, dass meine Bestrafung wegen intellektueller Urkundenfälschung erfolgt, wenn sich die obigen Angaben als falsch erweisen sollten.

16211

v. g. u. **Der Lagerkommandant**

Weisz József

KL/42/4.43 500.000

R EKG

Konzentrationslager .... Art der Haft: .... Gef. Nr.: 32554 ✓

Name und Vorname: Gafurow Turgunbaj ✓

geb.: 10. 1. 1916 zu: Klitschli, Kr. Katta-Kurgan, Pr. Samarkand, Uzb. S.S.R.

Wohnort: Klitschli, w. o.

Beruf: Landarbeiter / Lehrer Rel.: mohamm.

Staatsangehörigkeit: U. d. S.S.R. Stand: verh.

Name der Eltern: Vater: Landarb. Gafur Hasimow, verst. 1920 in Klitschli Mutter: Kondos G., geb. unbkt. verst. 1925 in Klitschli Rasse:

Wohnort: —

Name der Ehefrau: Fatima Bajzakowa Rasse:

Wohnort: Klitschki, w. o.

Kinder: 2/8-5 J. Alleiniger Ernährer der Familie oder der Eltern:

Vorbildung: Volksschule u. Hauptschule

Militärdienstzeit: .... von — bis

Kriegsdienstzeit: Artillerie, 1 M. von — bis 1941

Grösse: 173 Gestalt: mittelkräftig Gesicht: länglich Augen: braun

Nase: gebogen Mund: gew. Ohren: klein Zähne: 1 fehlt

Haare: schwarz Sprache: tadshik., russ. u. uzbek.

Ansteckende Krankheit oder Gebrechen:

Besondere Kennzeichen: keine

Rentenempfänger:

Verhaftet am: 11. 10. 1943 wo: Borisow

1. Mal eingeliefert: 21. 6. 1944, K.L. Bu. 2. Mal eingeliefert:

Einweisende Dienststelle: Sipo. Münster

Grund:

Parteizugehörigkeit: — von — bis

Welche Funktionen:

Mitglied v. Unterorganisationen:

Kriminelle Vorstrafen: —

Politische Vorstrafen: —

35.117

Ich bin darauf hingewiesen worden, dass meine Bestrafung wegen intellektueller Urkundenfälschung erfolgt, wenn sich die obigen Angaben als falsch erweisen sollten.

v. g. u. **Der Lagerkommandant**

Тургунбай Гафуров

KL/42/4.43 500,000

Control condition – neutral: Study 2

For Study 2 we adopted a neutral control condition. Participants in the control condition immediately answered outcome questions and demographics.

Section C: Ethics

Conducting research with conflict archives involves several ethical challenges. Conflict archives can provide unique evidence about violence and past injustice (Balcells and Sullivan, 2018). However, archival records in conflict archives tend to be shaped by those in power who were often the perpetrators (Skarpelis, 2020). Dealing with large quantities of victim files furthermore risks seeing the victims as data rather than individuals (Luft, 2020). We took steps to address these and additional concerns.

We conducted the research in close collaboration with our practice partner, the Arolsen Archives. Over the course of three years, we developed the research in conversation with the directorate and the education team, from defining research questions that are relevant to practice as well as academic scholarship, to crafting outcome measures that adequately captured the objectives of the #everynamecounts project, to discussing ethical concerns. In addition, all research team members filled out and then discussed the positionality questionnaire for field experiments by (Davis and Michelitch, 2022) to reflect on our identities and how they relate to the objectives of the project.

A first ethical concern relates to the emotional effect the archival files may have on participants. Seeing the original files documenting NS persecution may lead to emotional overwhelm. This is most pronounced in the information-only condition of Study 1, where participants receive potentially disturbing information about NS injustice without an opportunity to take meaningful action. We made sure that all information participants received was fact-based and commensurate to what they might read in a newspaper article or see in a museum.

A second concern relates to the trade-off between privacy and transparency. Our study uses historical files about individual victims, many of whom were brutally murdered based on the information in those files. None of the victims ever consented that these files would be accessible to the public, and we cannot know their preferences today. Here, we followed the lead of the Arolsen Archives. The Arolsen Archives, overseen by an international commission comprising 11 countries including Israel, have regularly reassessed their purpose and approach since their foundation soon after WWII. Since 2007, they have prioritized openness to the public, making their collections available to researchers and descendants of victims and survivors and the general public. The decision to digitize the archive and make it available online, as well as to engage people in this digitization effort via a crowd-sourced project makes the documents even more widely accessible. This should make it easier for descendants to find out about what happened to their ancestors and is seen as building a digital memorial. As such, our research does not violate the privacy of the victims beyond how their information is already handled, and for well-thought-through reasons. For more information about the decision to open up the archive, see "A Paper Monument: The History of the Arolsen Archives"(2019).

A third concern relates to honoring the victims and avoiding that they are perceived as mere data. The project is called #everynamecounts because by reading files with the names of victims, the often-nameless victims are remembered for a moment. Thus, while project participants do see several files, originally compiled by the NS regime, with limited information about the individuals, all files include the victims' names. Remembering their names is part of the purpose of the project and a form of commemoration. This is also highlighted in the instructional video shown in the treatment condition before digitizing (and one of the reasons why we included the video in the study).

A fourth concern was that participants treat the documents with care. It was paramount to us that participants take the task of digitizing seriously. In the information-only condition, we mentioned that the two scans participants saw were original files about actual NS victims and asked them to treat these documents with respect. In the digital history condition, we emphasized the importance of digitizing. After all, participants took part in a real digitization project, not a mock one. Our partner organization somewhat assuaged this concern: Every file in #everynamecounts is entered by multiple volunteers, so that even if some of our research participants made improper entries this will not negatively affect the record. To respect the victims and their family members it is also important that none of the historic files are abused, e.g., by ridiculing them or denying their authenticity. In our study, we present the files contextualized in the same way as they are contextualized by the Arolsen Archives. Since starting the crowd-sourcing effort, the archives have not observed any abuse (e.g., by far-right actors), even though the project has received a fair amount of publicity already.

Section D: Pre-registration

Research presented in this paper is preregistered. Below, we offer brief information on pre-analyses plans and explain a few, minor deviations from the plans.

Pre-analysis plans

Study 1 is preregistered at `https://osf.io/p892g`. Study 2 is preregistered at `https://osf.io/4u3sm`. Analyses were pre-specified before data collection. All the analyses reported in the main text are preregistered. For further information, please visit the provided links.

Deviations from the pre-analysis plans

In this paper, we adhered to pre-analysis plans with a few deviations that are detailed below.

Deviations from the pre-analysis plan in Study 1:

- *Number of observations:* We preregistered that we would recruit 600 participants, but only managed to recruit 552 participants. In the planning stage, the managers of the university laboratories suggested that we would be able to recruit up to 600 participants, possibly less. Neither of the laboratories has managed to fully replenish their participant pool since the Covid-19 pandemic. Over the course of two months, we advertised the study four times, with fewer new participants signing up each time.

Overall we were able to recruit 552 participants before exhausting all resources. Not being able to recruit 600 participants was thus beyond our control.

- *Presenting social norms:* In the pre-registration, we list social norms variables as outcomes. Feedback and discussion with other scholars made us realize that social norms are a process rather than an outcome. Thus, in the main paper, we report them as processes as opposed to outcomes.

- *Presenting prejudice:* We preregistered that digital history participation should reduce prejudice. Inconsistent with this hypothesis, we did not observe any significant effects on prejudice across studies. We report these findings in detail in the supplemental materials, section E.

- *Presenting pre-post effects:* Participants in both conditions first filled out an online questionnaire. A week later, participants came to the physical lab, were assigned to treatment or control conditions, and filled out another questionnaire. Both questionnaires included questions on outcomes and processes. As pre-registered in the main analysis, we compared participants in the treatment condition to participants in the control condition using the outcomes from the second survey while controlling for the outcomes from the first survey. We also preregistered that we would examine the pre-post effects in the treatment condition (i.e., comparing responses at time 2 to responses at time 1 in the treatment condition). Because these results have important limitations, we describe them in the supplemental materials (section F) instead of the main manuscript.

- *Heterogeneous effects by migration status:* In between data collection and analysis for Study 1, we realized that about half of the sample are people with a migration background (i.e. at least one parent was born outside of Germany). Given the lack of intergenerational ties to the Nazi past, we preregistered that treatment effects would likely be smaller for participants with a migration background than for participants without a migration background. This was not in the pre-analysis plan registered at `https://osf.io/p892g` but was registered after data collection and before data analysis at `https://osf.io/xce24`. The analysis revealed that except for collective guilt, there are no significant differences by migration background. For collective guilt, different from our expectation, the treatment has larger positive effects among participants with a migration background than among those without a migration background. Given the shortage of significant effects, we do not report these results further.

Deviations from the pre-analysis plan in Study 2:

- *Number of observations:* We pre-registered that we would recruit 1000 participants via Clickworker, a crowdsourcing platform for digital workers, but managed to recruit only 900 participants. We recruited participants between 22nd of November and 11th of December 2023. We managed to recruit 900 participants after advertising the study multiple times throughout this period. We could not recruit more participants after this date because the pool of willing participants was exhausted and we risked running into the Christmas holidays with the time 2 survey, which took place two weeks later.

- *Presenting social norms:* As in Study 1, we present social norms as a process rather than an outcome/dependent variable .
- *Presenting prejudice:* As in Study 1, we do not find any support for our preregistered hypothesis that digital history participation would reduce prejudice. We report these findings in the supplemental materials, section E.
- *Presenting behavioral outcomes:* Our main behavioral measure was preferences for donating to different organizations, including a Holocaust remembrance site and an anti-racism foundation. This measures was used in Study 1 and 2 and results are presened in the main text. We preregistered analyses related to the effects of participating in #everynamecounts on additional behavioral outcomes that were only included in Study 2 two-three weeks after the intervention. We did not observe any significant effects on behavioral outcomes. Instead of reporting them in the main text, we report them in the supplemental materials, section L.
- *Heterogeneous effects by party preferences:* We preregistered analyses on heterogeneous treatment effects by party preferences and analyses excluding participants on the very left side of the ideological spectrum. We report the results in the supplemental materials section I rather than the main text.

Section E: Effects on prejudice

In addition to the effects on mobilization for better intergroup relations, lower levels of prejudice could also contribute to better intergroup relations today. In fact, the psychological literature on interventions to improve intergroup relations primarily focuses on prejudice reduction (Brauer, 2024). We thus pre-registered that participation in a digital history project should reduce prejudice in Studies 1 and 2, in addition to the effects on mobilization reported in the main manuscript. We do not report these findings in the man manuscript because measuring prejudice in the context of original historical material of identity-based genocide is difficult, especially in the specific cultural context of Germany. Given the null effects, we prefer not to devote extensive space to the measurement strategy in the main manuscript

However, the prediction is consistent with a growing literature on systemic racism in the US, suggesting that a critical understanding of historical as well as other systemic injustices is linked to lower outgroup bias among Whites today (Banaji et al., 2021; Bonam et al., 2019). It is also in line with the policy goal that Holocaust education is supposed to improve intergroup attitudes today (UNESCO, 2017). If the participatory #everynamecounts intervention is effective at engaging people with the history of NS persecution and oppression (as it should be based on reasons we discuss in the main manuscript), it may enhance people's critical understanding of this history, and thus reduce their prejudice.

Study 1

Measuring prejudice in the context of original historical material of identity-based genocide is difficult. Highlighting the contextual nature of social categories (Cikara et al., 2022) our partners and we worried about using group categories in questionnaires right after participants see historical documents displaying the same or similar group categories used by the Nazis to organize repression and genocide. We were concerned that primed with the genocide, participants might interpret the categories in biological instead of social terms, and also find them more offensive than in other contexts. This concern was aggravated by the German context where people are – precisely due to the history of the Holocaust – generally more reluctant to use group categories (Juang et al., 2021). To resolve this dilemma, we measure prejudice with a traditional measure that ensures comparability across studies – the feeling thermometer – supplementing it with two measures specific to the German context, namely objection to political correctness, and objection to group categories. For the feeling thermometer, we asked how cold vs. warm participants feel towards 4 minoritized groups (Jews, Turkish-Germans, refugees, Roma and Sinti) on a scale from 1-100 and then formed an overall index aggregating across minoritized groups ($\alpha = 0.82$). For a German-specific scale of prejudice, we adopted the "rejection of political correctness" scale that shows relatively high mean values in representative surveys conducted by the German Racism Monitor (DeZIM, 2022). It is an aggregate of 5 items ($\alpha = 0.92$), with higher values representing higher objections to political correctness, e.g., "It is absurd that one is accused of racism when one merely asks where someone comes from". To assess concerns participants may have about the use of group categories, we adopted a measure of endorsement of colorblind ideology (Knowles et al., 2009) to the German context, aggregating across 3 items ($\alpha = 0.91$),

e.g., "I wish people in this society would stop obsessing so much about group categories". The list of all prejudice items are reported in Table S3.

Table S3: List of Prejudice Items

Rejecting Political Correctness *(Cronbach's* $\alpha = 0.92$*)*
Please read through the following statements and indicate whether you agree with them. (1 – Strongly disagree and 7 – Strongly agree).

Today, one is labeled a racist for every little thing.
Freedom of speech is restricted by political correctness.
It is an impertinence when historical figures, such as Martin Luther or Kant, are now accused of racism, sexism or antisemitism.
It is absurd that one is accused of racism when one merely asks where someone comes from.
It is nonsense that previously normal words should now be offensive.

Colorblindness *(Cronbach's* $\alpha = 0.91$ *in Study 1 and* 0.91 *in Study 2)*
Please read through the following statements and indicate whether you agree with them. (1 – Strongly disagree and 7 – Strongly agree).

I wish people in this society would stop obsessing so much about group categories.
People who become preoccupied by group categories are forgetting that we are all just human.
Putting group labels on people obscures the fact that everyone is a unique individual.

Feeling Thermometer *(Cronbach's* $\alpha = 0.79$ *in Study 1 and* 0.82 *in Study 2)*
How cold vs. warm do you feel towards following groups (0 – very cold and 100 – very warm)?

German-Turks
Jews
Refugees
Roma and Sinti

Contrary to our predictions, there are no significant effects on prejudice outcomes. The results are reported in Table S4. There is no evidence that participating in a digital history project compared to receiving information-only improves participants' feeling towards minoritized groups, decreases their rejection of political correctness, or changes their position towards the use of group categories as measured by the colorblindness index.[3]

Table S4: The effects of digital history participation on prejudiced beliefs in Study 1

Outcome	Coefficient	Std. Error	P-value	N
Rejecting political correctness index	0.03	0.03	0.41	552
Colorblindness index	-0.09	0.05	0.08	552
Feeling thermometer index	0.02	0.05	0.73	552

* $p < 0.05$, ** $p < 0.01$, *** $p < 0.001$

[3]In Study 1, the p-value for the effect of our treatment on colorblindness is 0.08. Although it does not reach the conventional 0.05 threshold, the treatment seems to affect opinions on colorblindness. Since in Study 2 the effect of treatment on colorblindness is close to 0, we do not further elaborate on this.

Deviating from our pre-registration, we repeat the feeling thermometer analyses separately for the different target groups: Turkish Germans, refugees, Jews, and Roma and Sinti. All groups are minoritized in Germany today, however, they had different positions during Nazi persecution, which might affect prejudice towards them. The results are reported in Table S5 and show no significant effects on feelings toward any of the minoritized groups.

Table S5: The effects of digital history participation on individual feeling thermometer items in Study 1

Outcome	Coefficient	Std. Error	P-value	N
FT toward Turkish Germans	1.09	1.65	0.51	552
FT toward Refugees	1.07	1.77	0.55	552
FT toward Jews	1.02	1.68	0.55	552
FT toward Roma & Sinti	-0.83	1.84	0.65	552

* $p < 0.05$, ** $p < 0.01$, *** $p < 0.001$

Unexpectedly, almost half of the sample in Study 1 has a migration background (i.e., at least one parent is born outside of Germany). This complicates the interpretation of the results, because some of the groups we ask about in the feeling thermometer questions may not technically be outgroups but rather part of the ingroup for some of our participants. Unfortunately, due to data privacy concerns we were not able to collect the exact origin country information, only whether the origin country is in the EU. While the coefficients change when we run the analyses excluding all immigrants or only non-EU immigrants (i.e., slightly larger effect sizes), the p-value for the treatment coefficient continues to be far from the conventional significance level, 0.05.

Study 2

Despite the null effects in Study 1, we pre-registered that participation in a digital history project should have an effect on prejudice in Study 2 because of the new control condition. Unlike the control condition in Study 1, the control condition in Study 2 does not present any information about the history of the Holocaust. We predicted that compared to not engaging in any form with the history of the Holocaust, the intervention might promote a more critical understanding of historical injustice, which might be linked to reduced prejudice. We measured prejudice with the feeling thermometer only (Cronbach's alpha = 0.82), and beliefs about group categories, as in Study 1 (Cronbach's alpha = 0.91). As in Study 1, and again in contrast to our predictions, there are no significant effects. Results are reported in Table S6. Similar to Study 1, there are no significant effects for either of the two outcomes. Participating in a digital history project compared to a neutral control condition does not improve participants' feeling towards minoritized groups, or change their position towards the use of group categories as measured by the colorblindness index. Examining participants' feelings towards individual minoritized groups in Table S7 reveals that the largest reduction of prejudice occurs for Jews, but the effect is not statistically significant.

Table S6: The effects of digital history participation on prejudiced beliefs in Study 2

Outcome	Coefficient	Std. Error	P-value	N
Colorblindness index	0.00	0.06	0.99	900
Feeling thermometer index	0.07	0.06	0.27	900

* p < 0.05, ** p < 0.01, *** p < 0.001

Table S7: The effects of digital history participation on individual feeling thermometer items in Study 2

Outcome	Coefficient	Std. Error	P-value	N
FT toward Turkish Germans	0.43	1.58	0.78	900
FT toward Refugees	1.22	1.69	0.47	900
FT toward Jews	2.63	1.59	0.10	900
FT toward Roma & Sinti	2.40	1.73	0.17	900

* p < 0.05, ** p < 0.01, *** p < 0.001

Discussion of Null Effects for Prejudice

Across two studies, using three outcome measures, we find no evidence that participating in a digital history project reduces prejudice. Several robustness checks suggest that this result is a meaningful null effect, especially given the large and persistent effects on some of our mobilization outcomes reported in the main manuscript. This is especially surprising in Study 2, where the bundled, digital history intervention is compared to a neutral control condition. It may be due to the format of how the information is presented in the digital history project. As discussed in the main manuscript, as a crowd-sourcing project that showcases how the Nazi machinery operated at a systemic level, the #everynamecounts intervention may be particularly effective for outcomes that tap into collective processes but less so for more individualized outcomes, such as prejudice and confronting discrimination (a mobilization outcome on the individual level). The null effect for prejudice combined with the positive effects for behavioral intention outcomes presented in the main manuscript also echos results from other large-scale intergroup interventions that find positive results for behaviors but not attitudes Mousa (2020); Paluck et al. (2021); Scacco and Warren (2018). Future research could investigate more systematically what formats of representing history can positively impact behavioral vs. attitudinal outcomes at the individual vs. collective level.

Section F: Within-subject effects in Study 1

The main objective of Study 1 was to compare the effect of digital history participation to receiving information only in a between-subjects study that took place in a physical laboratory. As described in Section A, we included a time 1 online assessment of our outcome variables in our statistical model to improve the efficiency of our time 2 treatment effect estimators.

In the pre-analysis plan, we also preregistered within-subject analyses. In other words, we planned to compare study participants' responses in the online questionnaire with their responses in the physical laboratory. This within-subject comparison can inform us about the effects of digital history participation when we limit the sample to participants in the digital history participation condition; and about the effects of exposure to information about history when we limit the sample to participants in the information-only control condition.

Within-subject comparisons are reported in Fig. S1. The left panel reports the results for the information-only condition and the right panel reports the results for the digital history condition. While we do not observe any significant change in collective action intentions for commemoration among participants in the information-only condition, there is a significant increase among participants in the digital history condition. We observe significant increases in collective action intentions for better intergroup relations in both conditions, but the effect is larger for participants in the treatment condition. Finally, there is a significant increase in intentions to confront discrimination in both conditions.

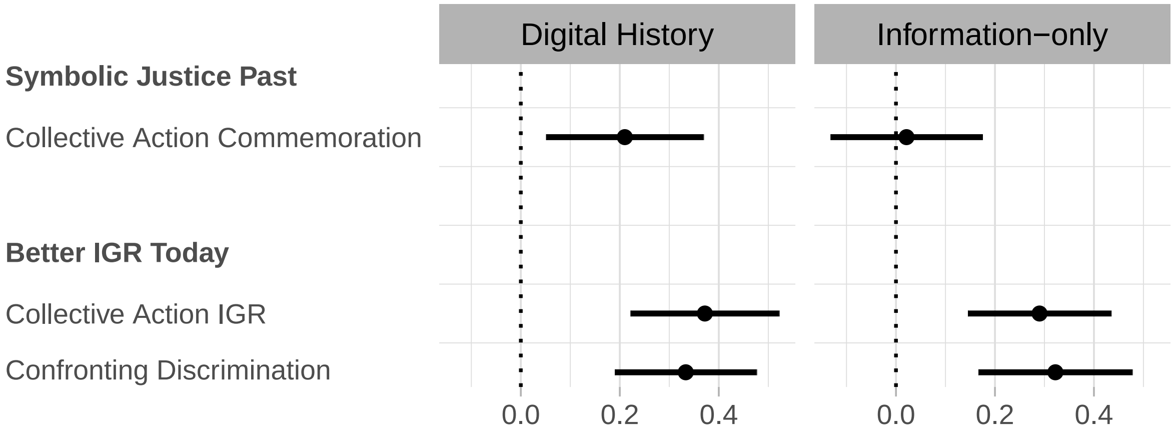

Fig. S1: Within-subject comparisons in Study 1. Dots denote standardized coefficients for treatment and bars to 95% confidence intervals.

While these results are mostly consistent with the results from the between-subjects analyses we report in Studies 1 and 2, a few issues stand out. Unlike past research (Balcells and Voytas, 2023; Leach et al., 2013), we do not observe defensiveness to the information participants receive in the information-only condition. This might be due to the strong social norm against defensiveness to information about Nazi persecution in Germany that was evoked by the pre-test. These activated social desirability concerns could also explain the increased intentions to confront discrimination that we do not observe between conditions

in Study 1 or Study 2. Overall, the design makes it difficult to explain the results of the within-subject analyses. After all, the main purpose of the online assessment was to improve our estimators. We thus fully rely on the between-subjects analyses from Studies 1 and 2 in the main manuscript.

Section G: Distribution of outcomes by condition

In the main manuscript we reported the treatment coefficients for our outcomes. In this section, we report boxplots of outcomes by condition, which offers more detailed information on the effect of treatment on outcomes. In Figure S2, we report the boxplots of mobilization intentions outcomes by condition in Study 1. Since donation variables are on a different scale than mobilization intentions outcomes, we report the boxplots of donation outcomes by condition in Study 1 in Figure S3. Similarly, we report the boxplots of mobilization intentions outcomes by condition in the first wave of Study 2 in Figure S4. Boxplots of outcomes for the second wave of Study 2 are reported in Figure S5 for mobilization outcomes and in Figure S6 for donation outcomes. While there are outliers for the confronting discrimination index, excluding these outliers does not impact our inferences. In the main manuscript, we concluded that there are null effects of the treatment on intentions to confront discrimination. When outliers are excluded from the analysis, the coefficient gets even closer to 0.

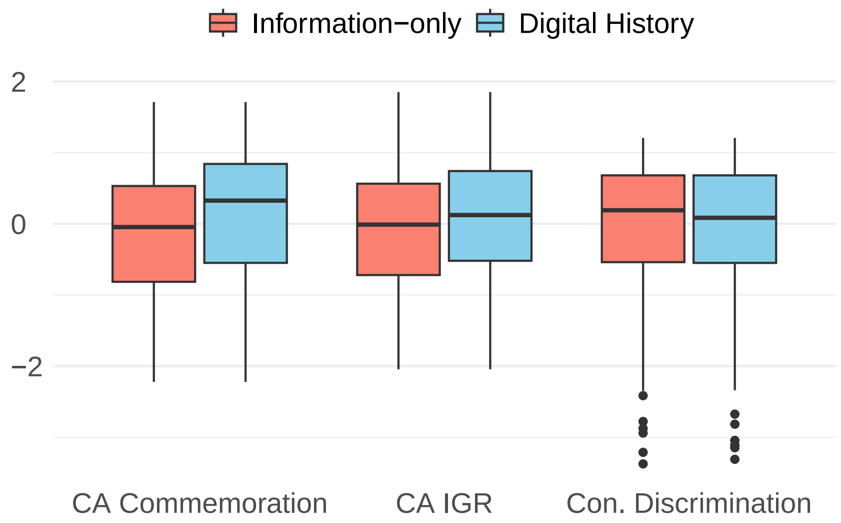

Fig. S2: Boxplots of mobilization intention outcomes by condition in Study 1

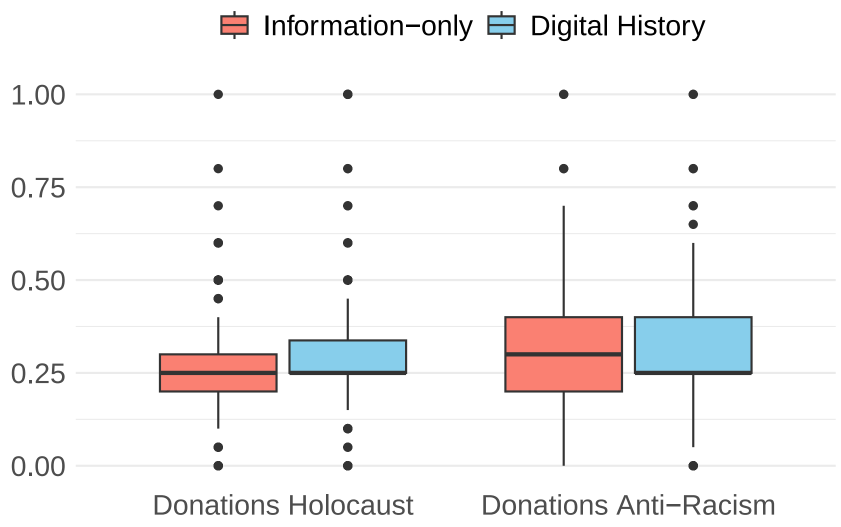

Fig. S3: Boxplots of donation outcomes by condition in Study 1

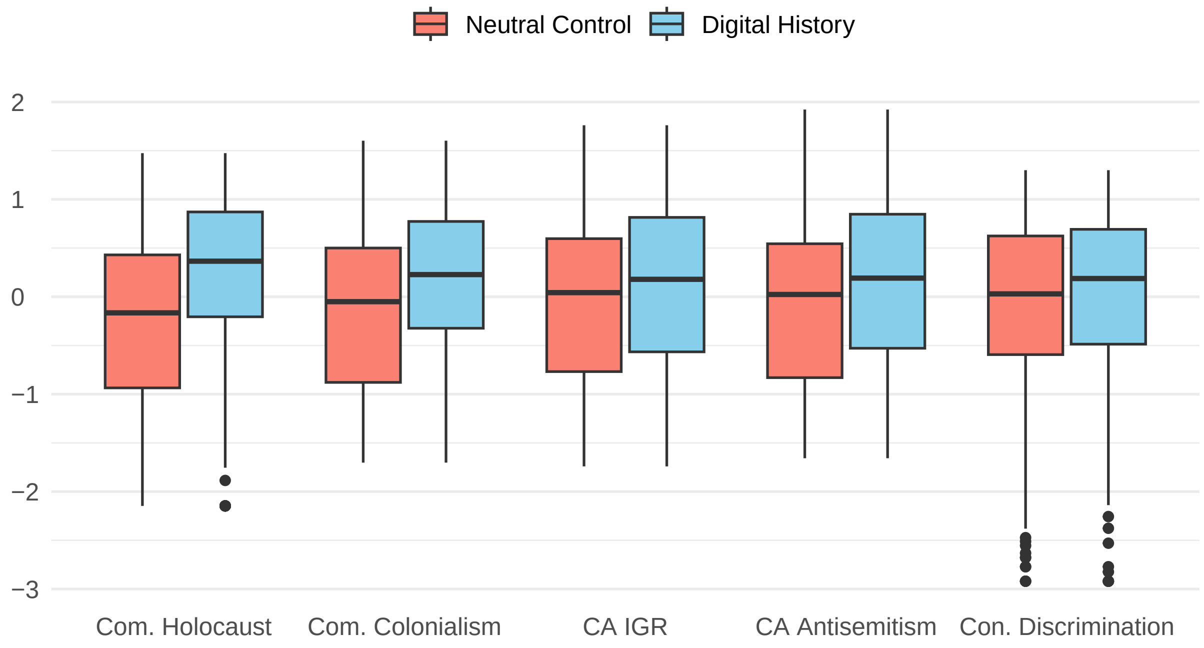

Fig. S4: Boxplots of outcomes by condition in the first wave of Study 2

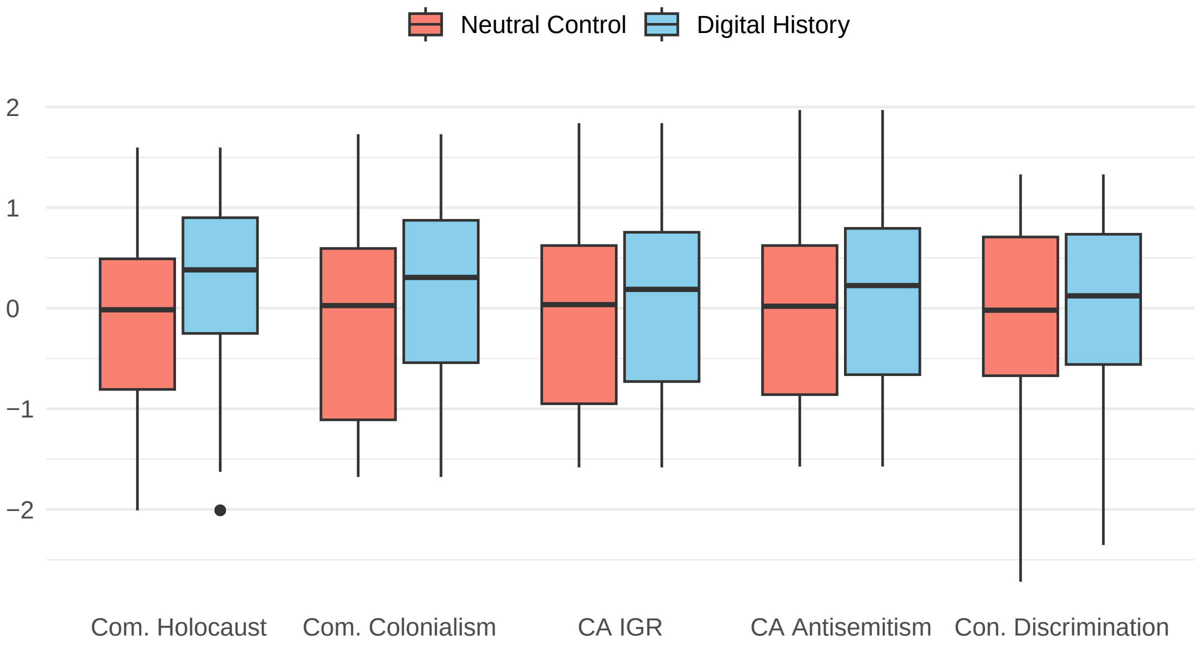

Fig. S5: Boxplots of mobilization intention outcomes by condition in the second wave of Study 2

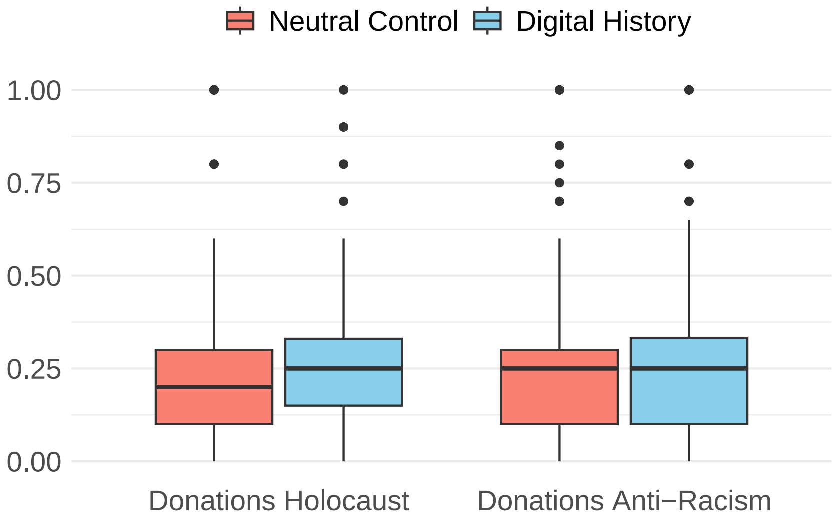

Fig. S6: Boxplots of donation outcomes by condition in the second wave of Study 2

Section H: Relations between outcomes and processes

In the main manuscript, we reported the effects of our treatment on outcomes and processes and suggest that participative efficacy beliefs are the main processes. In this section, we further illuminate the relationship between efficacy beliefs and outcomes by providing correlations between them. Table S8 reports correlations for Study 1 by condition. Participative efficacy shows the strongest positive correlation with intentions to engage in collective action for commemoration and collective action for better intergroup relations, suggesting that individuals with a high sense of efficacy are more likely to want to engage in those actions. Confronting discrimination is also highly correlated with efficacy. Donations, both for Holocaust-related and anti-racism causes, exhibit generally lower correlations with efficacy. All correlations except for donations for a Holocaust memorial site by participants in the digital history condition are statistically significant at the 0.05 level.

Table S8: Correlations between participative efficacy and outcomes in Study 1

	Information-only	Digital History
Collective action commemoration	0.62	0.63
Donations Holocaust	0.23	0.12
Collective action IGR	0.55	0.56
Confronting discrimination	0.37	0.47
Donations anti-racism	0.23	0.13

Table S9 reports correlations between outcomes and processes for the first wave of Study 2. In general, all three types of efficacy indices show high correlations with outcomes. While participative efficacy consistently shows the strongest correlation, group efficacy has a somewhat less pronounced but still meaningful correlation with our outcomes of interest. Correlations are stronger among participants in the digital history condition. Among all outcomes, efficacy indices have the strongest correlation with the commemoration Holocaust index. All correlations are statistically significant at the 0.05 level.

Table S9: Correlations between type of efficacy and outcomes in the first wave of Study 2

	Neutral Control			Digital History		
	PE	SE	GE	PE	SE	GE
Commemoration Holocaust	0.46	0.39	0.33	0.61	0.55	0.53
Commemoration Colonialism	0.39	0.33	0.19	0.53	0.45	0.45
Collective action IGR	0.41	0.35	0.21	0.42	0.41	0.39
Collective action antisemitism	0.40	0.36	0.27	0.45	0.43	0.42
Confronting discrimination	0.51	0.38	0.33	0.55	0.43	0.45

PE = Participative efficacy, SE = Self efficacy, GE = Group efficacy

Section I: Heterogeneous treatment effects by party preferences in Study 2

Since reactions to the remembrance of past atrocities may vary based on people's party preferences, we pre-registered an analysis of heterogeneous treatment effects by party preferences in Study 2. In the sample, 200 participants indicated that they would vote for the CDU/CSU (Christian Democrats), 192 for Bündnis 90/Die Grünen (Greens), 114 for the SPD (Social Democrats), 92 for the AfD (far right), 84 for the FDP (Liberals), 66 for Die Linke (Left), and 148 for others. We split the sample and ran the same analysis as in the main analysis. The results are reported in Fig. S7. Given sample size limitations by subgroup, the point estimates are important.

It is encouraging that the effects of participating in #everynamecounts on collective action intentions for commemoration are consistent across parties. They are particularly large for supporters of the CDU/CSU (Christian Democrats). There is, however, variation in the effects on collective action intentions for better intergroup relations. While the treatment has substantial effects among Greens (Bündnis 90/Die Grünen), Christian Democrats (CDU/CSU), and Left (die Linke) voters, the effects are smaller among supporters of the far right (AfD), Liberals (FDP) and Social Democrats (SPD). Despite this variation in coefficient sizes, we do not observe statistically significant differences between parties.

We also preregistered to repeat the analysis by excluding participants who self-reported as 0-2 on a political ideology scale (0-left and 10-right). The results are reported in Fig. S8 and suggest no difference in effects based on far-left political ideology.

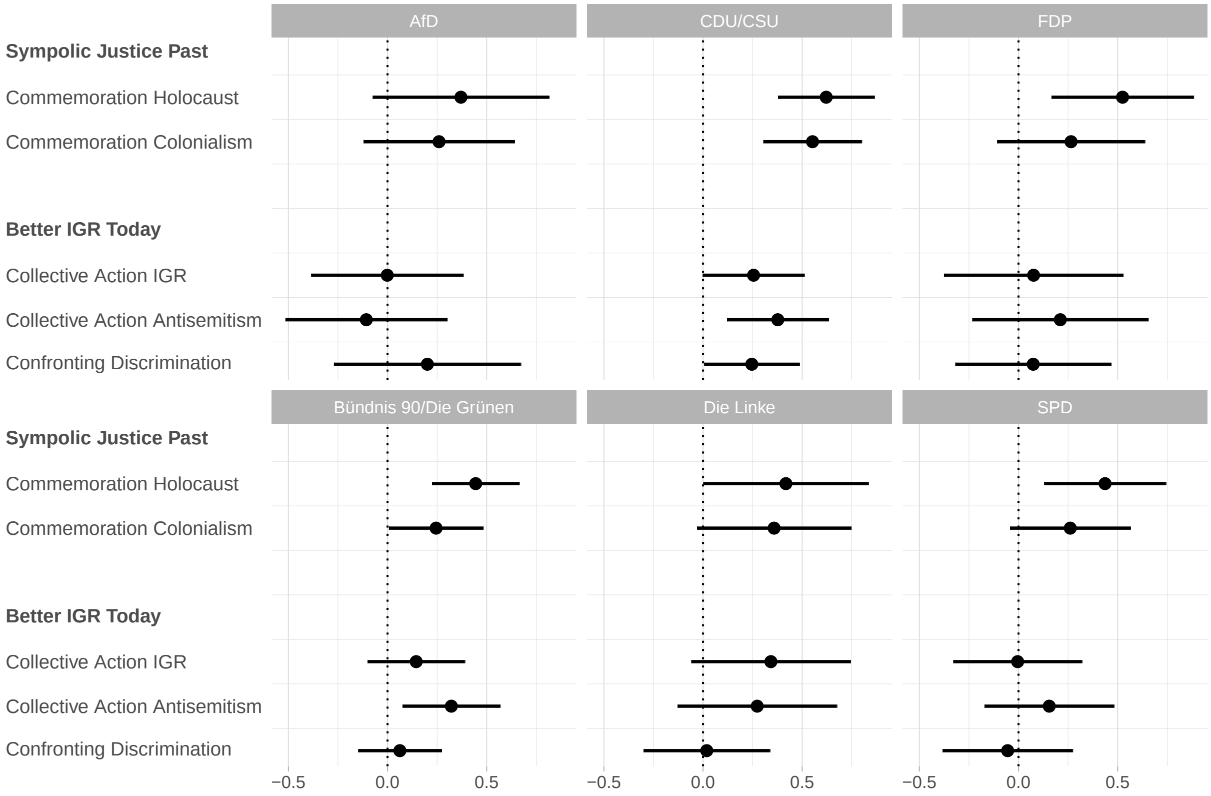

Fig. S7: The immediate effects of participating in #everynamecounts by party preferences. Dots denote standardized coefficients for treatment and bars to 95% confidence intervals. N= 752. AfD N= 92, CDU/CSU N= 200, FDP N= 84, Bündnis 90/Die Grünen N= 192, Die Linke= 66, and SDP N= 114. People who do not support a party, do not know which party to support, and support another party are excluded from the analysis.

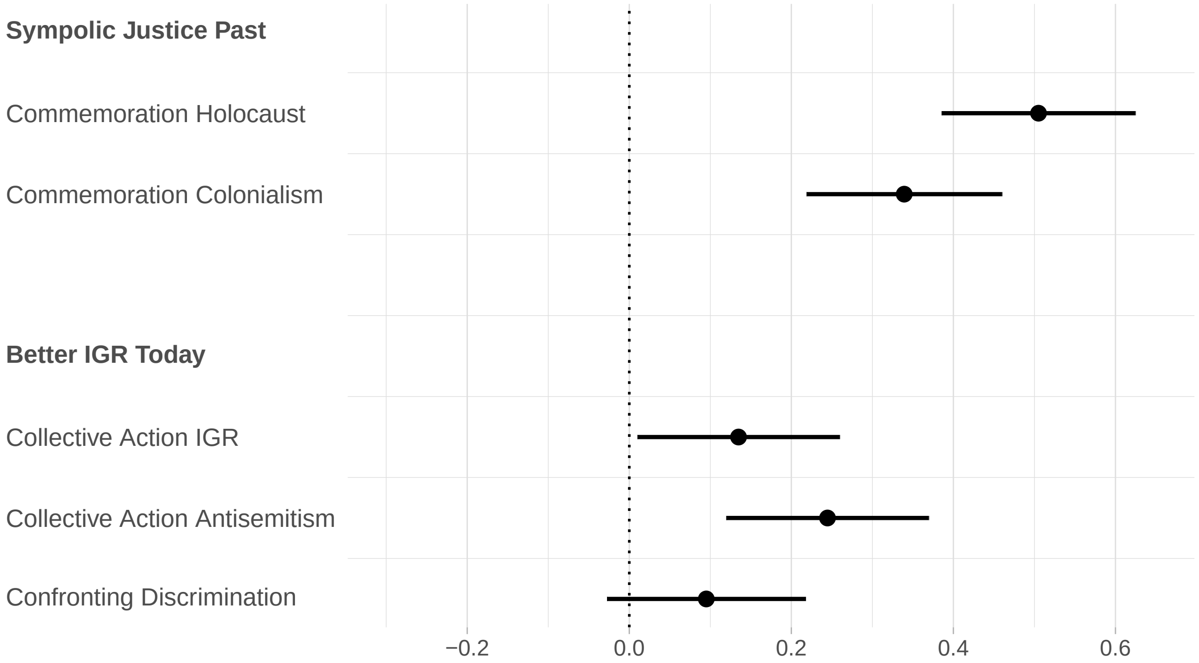

Fig. S8: The immediate effects of participating in #everynamecounts when participants who self-reported as 0-2 on a political ideology scale (0-left and 10-right) are excluded. Dots denote standardized coefficients for treatment and bars to 95% confidence intervals. N= 783.

Section J: Selective attrition in Study 2

The design in a digital field setting, as opposed to a laboratory setting, bears a risk for selective attrition. Participants in the digital history condition had to access our partner's platform independently and perform a complex task (digitizing archival documents, which are often handwritten), while participants in the control condition only had to fill out a simple questionnaire. Indeed, more people dropped out in the digital history than in the control condition, as reported in Table S10. However, an analysis using Lee bounds confirms the robustness of most of our findings.

Table S10: Attrition in Study 2

Condition	N Assigned	N Finished	N Dropped
Neutral Control	509	490	19
Digital History	512	410	102

Unfortunately, we do not know why digital workers dropped out selectively in the digital history condition. There are many possible reasons. For example, digitizing historical documents takes between 15 and 20 minutes and requires a great amount of sustained attention. Some digital workers on the Clickworker platform may prefer shorter or less complex tasks. Some participants may also be sensitive to content that features violence and prefer lighter content. Others may have a personal or family trauma that is activated when engaging with the history of the Holocaust. Yet others may be opposed to remembrance of the Holocaust because they are antisemitic, racist, or otherwise prejudiced or because they want to distance themselves from the past. Because we do not know the reasons for attrition, we adopt a cautious approach and carry out additional analyses to investigate the robustness of our results where we make more and less conservative assumptions about dropout.

To create transparency about the robustness of our results we use the trimming approach suggested by (Lee, 2009). Simply put, to calculate Lee bounds, we trimmed 80 observations from the group with less attrition, the control condition, to create equal group sizes. We then estimated treatment coefficients as in the main text but based on this smaller sample. We ran these analyses twice for important outcomes, once based on a sample where we trimmed the 80 participants with the lowest values on the outcome variable (i.e., left bound) and once based on a sample where we trimmed the 80 the participants with the highest values (i.e., right bound). The results are reported in Fig. S9.

Both bounds make conservative assumptions but in different directions. The right bound (the dot on the right) shows the treatment effect when the observations trimmed in the control condition were those with the 80 highest values for the respective outcome. For example, for intentions to commemorate this assumes that all participants who dropped out in the digital history condition where the ones with the highest intentions to commemorate. This might be because they have a strong connection to the victims and therefore find the digital history condition traumatic. The left bound (the dot on the left) shows the treatment effect when the observations trimmed in the control condition were those with the 80 lowest values for the respective outcome. For example, for intentions to commemorate this assumes that all participants who dropped out in the digital history condition were the ones with the

lowest intentions to commemorate. This might be because they want to move on from the past and see no value in commemoration.

Fig. S9 shows that most of our results are robust even to the most conservative assumption that all 80 participants dropped out for the same reason. Specifically, this is true for intentions to commemorate and efficacy beliefs. The results are less robust for intentions to fight antisemitism and act in support of better intergroup relations today. If we assume that all participants dropped out because of their extremely low values on the respective outcome variable, the treatment effect crosses zero and even changes direction.

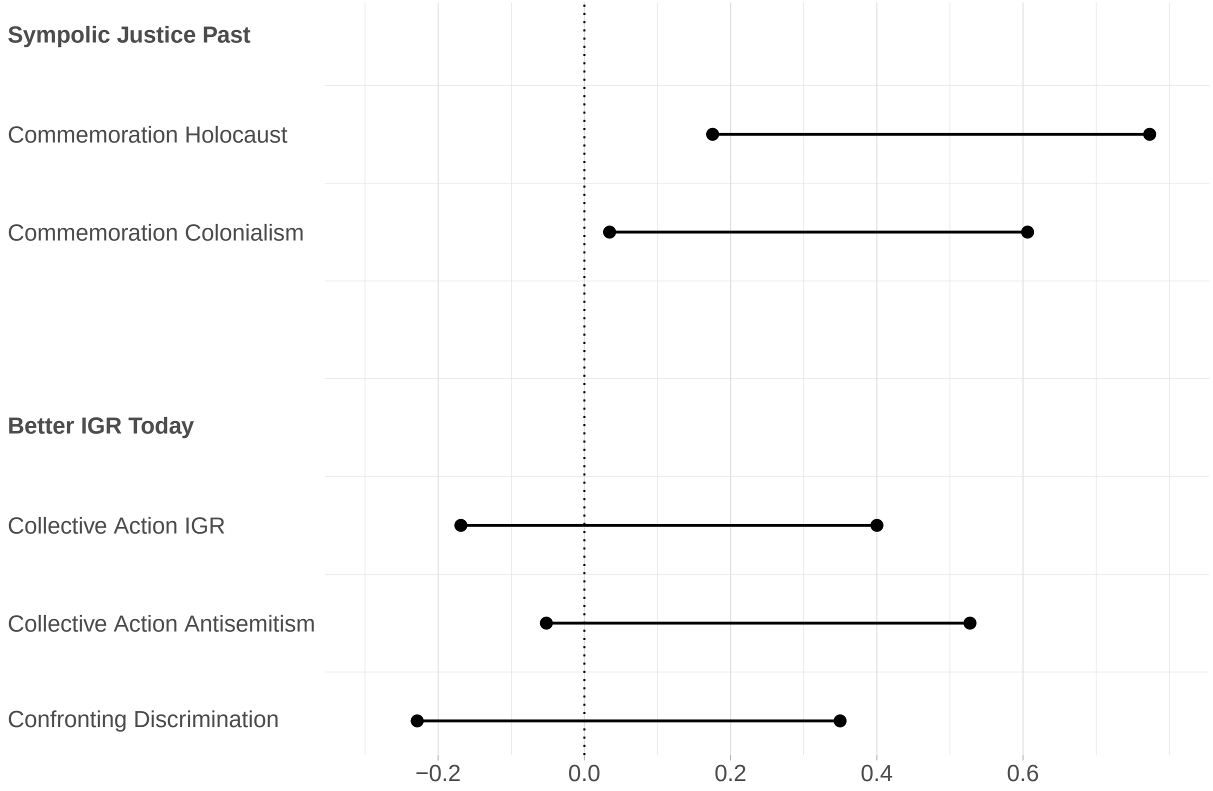

Fig. S9: Lee bounds of the immediate effects of participating in #everynamecounts.

As mentioned above, Lee bounds represent a conservative assumption. They assume that all dropouts are participants with either the lowest or highest values on the relevant outcome variable. While this is certainly plausible in some cases, it might not be true for all dropouts considering the many possible reasons for dropping out discussed above. To create transparency about the assumptions under which our treatment effects are robust to selective attrition, we carried out an analysis that can be considered a sensitivity check for the lower bound Lee estimate. Instead of trimming the 80 lowest values for a given outcome in the control condition, we trimmed a combination of the lowest values and a random subset. We started the analysis by trimming the lowest 78 observations and two random observations, followed by trimming the lowest 77 observations and three random observations until we had trimmed all 80 observations at random. For each combination of random and systematic trims, we repeated the analysis 1000 times to obtain the range of

possible treatment coefficient estimates under each assumption. The results are reported in Fig. S10. The X-axis denotes the number of observations that are trimmed at random and the Y-axis denotes the treatment coefficients obtained in each of the 1000 analyses.

The results for intentions to fight against antisemitism suggest that when we trim 61 observations with the lowest values and 19 observations at random, all treatment coefficient estimates are positive. To observe consistently positive treatment coefficient estimates for intentions to support better intergroup relations, we have to assume that 56 observations are trimmed at random. Overall the Lee bounds analyses show that results for symbolic justice and efficacy beliefs are remarkably robust. Results for better intergroup relations today are fairly robust for fighting antisemitism but require stronger assumptions about random dropouts for intentions to take collective action in support of better intergroup relations today.

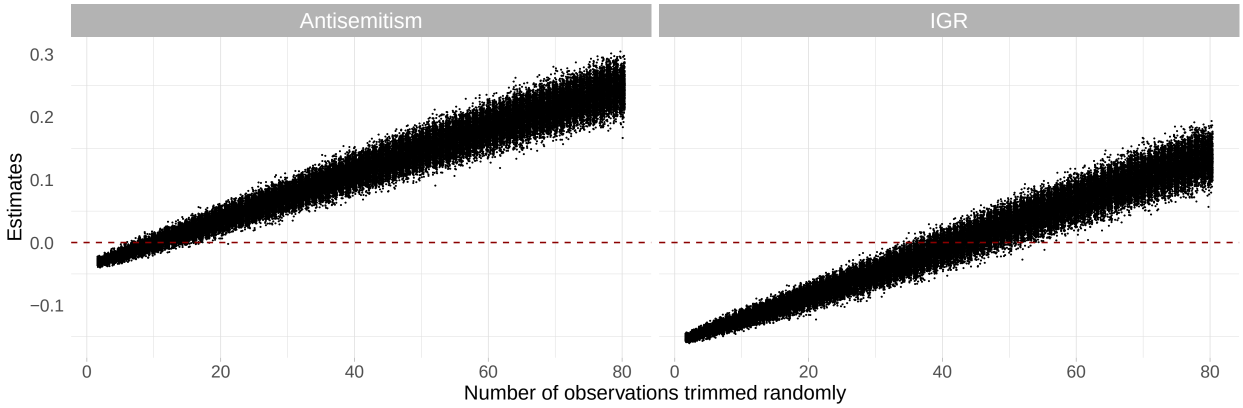

Fig. S10: Sensitivity check for the lower Lee bounds of the immediate effects of participating in #everynamecounts.

Section K: Limiting the sample to those who attended both surveys in Study 2

In the main text, for Study 2 we reported the results for all participants at time 1 (N=900) and at time 2 (N=487). We examined changes in the coefficient sizes between the two time points to assess the impact of participating in #everynamecounts over time. In this section, as a robustness check, we limit the sample to those who completed our survey at both time points and repeat the analyses from the main text. The coefficients are reported in Table S11. The results are similar to the ones reported in the main text and - especially considering that we almost halved our sample size for these analyses - highlight the robustness of our main findings. For the most part, treatment effects are still significant at time 2. Where they are not significant (collective action intentions for better intergroup relations) they are still positive in the same direction as at time 1.

Table S11: The effects of digital history participation on mobilization compared between time 1 and 2. The sample is limited to those who attended both surveys.

Outcome	Time 1	Time 2
Commemoration Holocaust	0.48	0.34
Commemoration Colonialism	0.22	0.34
Collective Action Antisemitism	0.27	0.19
Collective Action IGR	0.17	0.13
Confronting discrimination	0.11	0.11

Section L: Behavioral outcomes in Study 2

In Study 2, we added the following new behavioral outcomes in the survey that participants completed two-three weeks after the intervention.

Behavior: Writing a letter

Following (Adida et al., 2018) we offered participants the opportunity to write an anonymous letter to the Federal President in support of vandalized Holocaust memorials. 107 participants wrote a letter and letters were on average 57 words long. The instructions read: "Recently, there has been an increase in attacks on concentration camp memorials by right-wing extremists. Cases of vandalism, graffiti and hostile statements are on the rise throughout Germany. Incidents are reported almost every week. We are collecting letters in which people speak out in support of memorials that have been vandalized or otherwise attacked. We will send anonymous letters to the Federal President to ask for his support." We then asked: Would you like to write a short, anonymous letter to the Federal President expressing your support for memorials that have been vandalized? (Yes/No) If participants clicked yes, a text field for the letter appeared.

Behavior: Interest in Stolpersteine

We gave participants an opportunity to learn more about a public commemoration project ("Stolpersteine" / "stumbling stones"). 30 people clicked on the link. The instructions read: "If you are interested in more projects on the topic of remembering Nazi injustice, you can click here to find out more about Stolpersteine for Nazi victims and survivors in your area: https://stolpersteine-guide.de/"

Behavior: Sharing #everynamecounts

We gave participants an opportunity to share their experience with others. Only one person clicked on the link to share it. The instructions read: "#everynamecounts is a project of the Arolsen Archives. At #everynamecounts, volunteers record the names and dates of victims and survivors of National Socialism so that descendants can find out what happened to their ancestors and so that the names are not forgotten. You too can take part and set an example for respect, diversity and democracy today. You can visit the project here [https://everynamecounts.arolsen-archives.org/]. If you would like to tell your friends or family about #everynamecounts, click here to share the link with them via WhatsApp [WhatsApp share button]."

Behavior: Donation As in Study 1, we gave participants an opportunity to divide up donations. The instructions read: "We will donate a total of 100 Euro to NGOs and participants can vote how we should divide up the amount between the following four organizations:

- KZ Gedenkstätte Dachau (concentration camp memorial site in Germany)
- Amadeu Antonio Stiftung (foundation against racism and anti-semitism today)
- Gesellschaft der Freunde der Akademie der Künste (foundation for art)
- NABU (environmental conservation organization)"

In Study 1, we observed a positive, immediate effect of digital history participation on a meaningful behavior: donating to a Holocaust memorial. In Study 2, we measured behavioral effects only two to three weeks after the intervention. When designing the study we faced a trade-off. If we asked behavioral questions, we would essentially offer opportunities for participation in the context of remembrance in the control condition. For example, donating to a Holocaust memorial could selectively strengthen the efficacy beliefs of those who donate, and thus bias any estimates of long-term effects of #everynamecounts. Since we already have evidence of immediate behavioral effects in Study 1, we prioritized measuring long term effects in Study 2. When examining the behavioral outcomes in Study 2 it is important to keep in mind that these were assessed two to three weeks after the treatment. The lack of significant effects here does not mean #everynamecounts does not affect behaviors. It rather means that #everynamecounts effects on behavior are limited in the long-term. Immediate positive effects on behavior are documented in Study 1.

Study 2 repeats the donation measure from Study 1 two-three weeks after the intervention together with several additional behavioral measures that are described above: writing a letter to the president, clicking on a link to learn about the Stolpersteine memorial, and sharing one's experience with #everynamecounts via WhatsApp. There are no statistically significant long-term effects on any of these behaviors. The results are reported in Table S12.

First, we asked participants to distribute donations among the same organizations as in Study 1. In Study 1, we observed a 4 EUR increase in donations to the Holocaust memorial immediately after taking part in #everynamecounts. In Study 2, we measured the effects two-three weeks later and still observed 2.7 EUR more donations. Although this effect is not statistically significant in the much smaller time 2 sample, it is quite substantive with more than 10% increase compared to the control condition.[4]

Second, we gave participants an opportunity to write a letter to the Federal President asking for his support of vandalized concentration camp memorial sites. Around 22% of participants wrote an anonymous letter to the Federal President but there are no differences by condition.[5]

Third, we offered information on Stolpersteine ("stumbling blocks" commemorating the victims of Nazi persecution). Only 6% of participants clicked on the link - perhaps Stolpersteine are already well known across Germany - and there were no differences by condition.

Fourth, we provided information about #everynamecounts as this was the first time participants in the control condition heard about it and suggested to all participants that they share this information via WhatsApp with their friends and family members. Of 487 participants, only one participant clicked on the share button, making an analysis impossible. This reluctance to share might be because of Germans' sensitivity to data privacy.

[4]There is no significant effect of the treatment on donations to the arts organization ($b = -0.01$, SE$= 0.02$, $p = .47$) or the environmental organization ($b = -0.03$, SE$= 0.02$, $p = .26$).

[5]None of the participants wrote anything negative.

Table S12: The effects of participating in #everynamecounts on behavioral outcomes two-three weeks after the intervention in Study 2

Outcome	Coefficient	Std. Error	P-value	N
Writing a letter	0.00	0.04	0.93	487
Interest in Stolpersteine	0.02	0.02	0.47	487
Donation: Holocaust Memorial	2.69	1.78	0.13	487
Donation: Foundation against Racism	1.31	1.83	0.47	487

* $p < 0.05$, ** $p < 0.01$, *** $p < 0.001$

References

Adida, C. L., Lo, A., and Platas, M. R. (2018). Perspective taking can promote short-term inclusionary behavior toward Syrian refugees. *Proceedings of the National Academy of Sciences*, 115(38):9521.

Balcells, L. and Sullivan, C. M. (2018). New findings from conflict archives: An introduction and methodological framework.

Balcells, L. and Voytas, E. (2023). What Difference Do Museums Make? In Meierhenrich, J., Hinton, A. L., and Douglas, L., editors, *The Oxford Handbook of Transitional Justice*. Oxford University Press, 1 edition.

Banaji, M. R., Fiske, S. T., and Massey, D. S. (2021). Systemic racism: individuals and interactions, institutions and society. *Cognitive research: principles and implications*, 6(1):82.

Bonam, C. M., Nair Das, V., Coleman, B. R., and Salter, P. (2019). Ignoring History, Denying Racism: Mounting Evidence for the Marley Hypothesis and Epistemologies of Ignorance. *Social Psychological and Personality Science*, 10(2):257–265. Publisher: SAGE Publications Inc.

Brauer, M. (2024). Stuck on Intergroup Attitudes: The Need to Shift Gears to Change Intergroup Behaviors. *Perspectives on Psychological Science*, 19(1):280–294. Publisher: SAGE Publications Inc.

Cikara, M., Martinez, J. E., and Lewis Jr, N. A. (2022). Moving beyond social categories by incorporating context in social psychological theory. *Nature Reviews Psychology*, 1(9):537–549.

Davis, J. and Michelitch, K. (2022). Introduction to field experiments: Thinking through identity and positionality. *PS: Political Science &; Politics*, 55(4):735–740.

DeZIM (2022). *Rassistische Realitäten: Wie setzt sich Deutschland mit Rassismus auseinander?* Deutsches Zentrum für Integrations- und Migrationsforschung DeZIM, Berlin.

Juang, L. P., Moffitt, U., Schachner, M. K., and Pevec, S. (2021). Understanding Ethnic-Racial Identity in a Context Where "Race" Is Taboo. *Identity*, 21(3):185–199. Publisher: Routledge _eprint: https://doi.org/10.1080/15283488.2021.1932901.

Knowles, E. D., Lowery, B. S., Hogan, C. M., and Chow, R. M. (2009). On the malleability of ideology: Motivated construals of color blindness. *Journal of Personality and Social Psychology*, 96:857–869. Place: US Publisher: American Psychological Association.

Leach, C. W., Zeineddine, F. B., and Čehajić Clancy, S. (2013). Moral Immemorial: The Rarity of Self-Criticism for Previous Generations' Genocide or Mass Violence: The Rarity of Self-Criticism. *Journal of Social Issues*, 69(1):34–53.

Lee, D. S. (2009). Training, wages, and sample selection: Estimating sharp bounds on treatment effects. *Review of Economic Studies*, 76(3):1071–1102.

Luft, A. (2020). How Do you Repair a Broken World? Conflict(ing) Archives after the Holocaust. *Qualitative Sociology*, 43(3):317–343.

Mousa, S. (2020). Building social cohesion between Christians and Muslims through soccer in post-ISIS Iraq. *Science*, 369(6505):866.

Paluck, E. L., Porat, R., Clark, C. S., and Green, D. P. (2021). Prejudice Reduction: Progress and Challenges. *Annual Review of Psychology*, 72(1):533–560. _eprint: https://doi.org/10.1146/annurev-psych-071620-030619.

Scacco, A. and Warren, S. S. (2018). Can Social Contact Reduce Prejudice and Discrimination? Evidence from a Field Experiment in Nigeria. *American Political Science Review*, 112(3):654–677. Publisher: Cambridge University Press.

Skarpelis, A. K. M. (2020). Life on File: Archival Epistemology and Theory. *Qualitative Sociology*, 43(3):385–405.

UNESCO (2017). *Education about the Holocaust and preventing genocide: a policy guide*. UNESCO, Paris.
